# Investigation of d-Amino Acid-Based Surfactants and Nanocomposites with Gold and Silica Nanoparticles as against Multidrug-Resistant Bacteria Agents

**DOI:** 10.1021/acsomega.2c04220

**Published:** 2022-12-08

**Authors:** Jae Ho Shim, Sungduk Gwak, Byung Kook Ahn, Hogyu Han, Yeonsun Hong, Ok Sarah Shin

**Affiliations:** † Department of Anatomy, Korea University College of Medicine, Seoul 02842, Korea; ‡ Department of Chemistry, Korea University, Seoul 02841, Korea; § Department of Microbiology and Immunology, David H. Smith Center for Vaccine Biology and Immunology, University of Rochester, Rochester, New York 14642, United States; ∥ BK21 Graduate Program, Department of Biomedical Sciences, College of Medicine, Korea University Guro Hospital, Seoul 08308, Korea

## Abstract

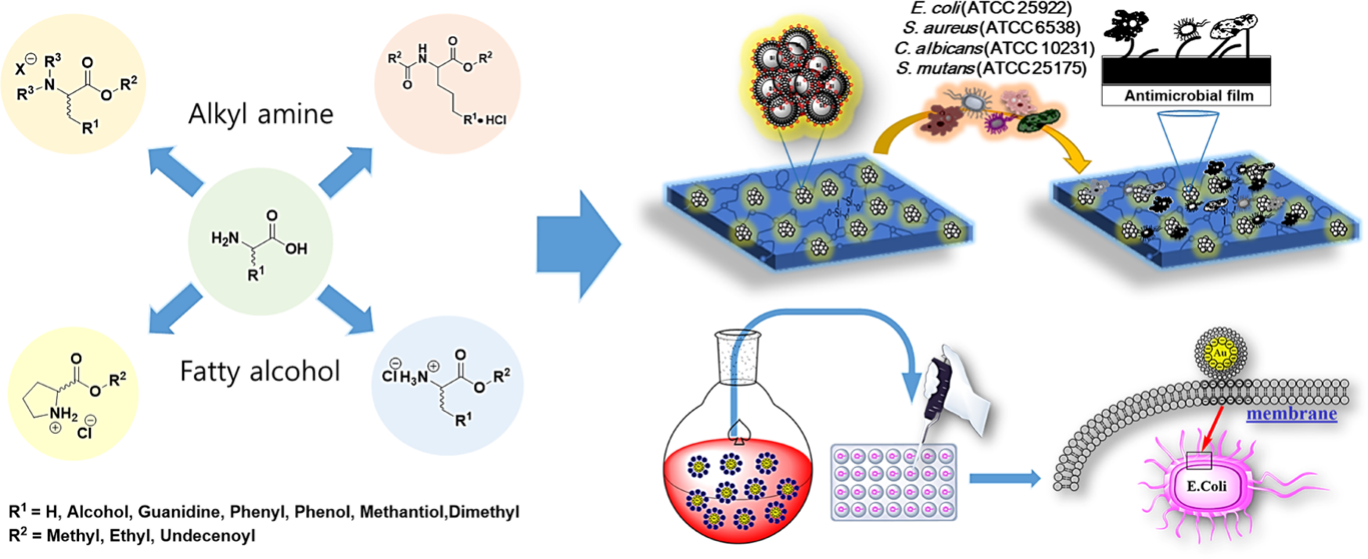

d-amino acid-based surfactants (d-AASs) were synthesized and their antimicrobial activity was evaluated. *N*-α-lauroyl-d-arginine ethyl ester hydrochloride (d-LAE), d-proline dodecyl ester (d-PD), and d-alanine dodecyl ester (d-AD) were found to have antibacterial activity against both Gram-positive and -negative bacteria, but less efficacy against Gram-negative bacteria. For these reasons, combining antimicrobial agents with nanoparticles is a promising technique for improving their antibacterial properties to eliminate drug-resistant pathogens. d-LAE coated on gold (AuNP) and silica (SiNP) nanoparticles has more efficient antibacterial activity than that of d-LAE alone. However, unlike d-LAE, d-PD has enhanced antibacterial activity upon being coated on AuNP. The antibacterial d-AASs and their nanocomposites with nanoparticles were synthesized in an environmentally friendly manner and are expected to be valuable new antimicrobial agents against multidrug-resistant (MDR) pathogens.

## 1 Introduction

The emergence of antimicrobial resistance is one of the major global public health problems in the 21st century, which has resulted in a robust increase in multidrug-resistant (MDR) pathogens against one or multiple antibiotics. Although several antibiotics have been used to effectively treat bacterial diseases, the abuse of antibiotics has led to the emergence of MDR bacteria.^1^ Pathogens adhere to surfaces and secrete polymeric substances to form biofilms that prevent the transfer of antimicrobial substances into the bacteria. This significantly increases the resistance of the bacteria to antimicrobial substances by a factor of thousands or more.^2^ Therefore, the inhibition of the early growth of bacteria before biofilm formation is crucial.

The contact-killing strategy involves the use of an antimicrobial agent to kill bacteria by slowly releasing inorganic substances such as metal (silver, copper, etc.) and nanoparticles (NPs) or organic substances such as antibiotics. This strategy also involves the immobilization of an antimicrobial agent on surfaces through a multistep chemical reaction. However, this strategy exhibits limitations, such as biotoxicity problems, low activity, and complexity in coating the antimicrobial agent and organic solvent. Therefore, there is a need to research and develop substances that combat various pathogens, including antibiotic-resistant bacteria, and that do not induce antibiotic resistance in an environment-friendly manner.

Antimicrobial peptide (AMP) is a defense-derived substance of the innate immune system that exists in all organisms and consists of about 10–60 amino acids, attracting attention as a potential alternative antibiotic.^3,4^ AMP, which has a low propensity to the development of resistance by microorganisms, shows high activity *in vitro*, but low activity *in vivo* often.^5^ Therefore, studies that introduce d-amino acids as one of the methods to solve this problem have been reported. It is known that d-amino acid peptides have a unique propensity to resist proteolytic cleavage as they self-associate within the bacterial membrane rather than enter the cytoplasm and interact with enzymes or receptors.^6^ There are many studies on the substitution of d*-*amino acids in AMP and their efficacy.^7^

Amino acid-based surfactants (AASs) are easier to introduce various functional groups (fatty alcohol, fatty acid, etc.) compared to AMP and have relatively high antibacterial activity and low production cost, so studies showing their potential as potential AMP candidates have been reported.^4,8^ As an antibiotic substitute, the antimicrobial amino acid-based surfactant (AAS) is a d-amino acid derived from an antimicrobial peptide and is present in all organisms as a defense-derived substance in the innate immune system.^3^ As most of these surfactants are positively charged and contain hydrophobic amino acid residues, they exhibit amphiphilicity. They kill bacteria either by destroying the cell membranes of negatively charged bacteria or by binding with the internal substances of the bacteria after penetrating their cell membranes. Thus, it is less likely to induce resistance in AAS compared with antibiotics that target specific receptors. Moreover, the rapid decomposition of AAS after action makes it safe when used in the body. As AAS acts on antibiotic-resistant bacteria, it could be a beneficial antibiotic substitute.^9^

*N*-α-lauroyl-l-arginine ethyl ester hydrochloride (LAE) is a cationic AAS derived from lauric acid, l-arginine, and ethanol. LAE has been incorporated into biopolymers to create their antimicrobial properties by resisting microbial adhesion and biofilm formation.^10^ Inclusion of LAE in films and particulates may reduce the interference of its antimicrobial effectiveness with anionic and hydrophobic components in foods and biosystems. Antimicrobial AASs have been coated on nanoparticles (NPs) to develop nanocomposites with enhanced antimicrobial efficacy. Nanocomposites based on silver nanoparticles (AgNPs) and tryptophan/tyrosine-based surfactants (TrpSs/TyrSs) showed antibacterial activity against both Gram-positive and -negative bacteria, which are known to be killed by TrpSs/TyrSs and AgNPs, respectively.^11^ Nanocomposites based on gold nanoparticles (AuNPs) and cysteine-based surfactants (CysSs) showed higher antitumor activity than CysSs alone due to the enhanced antitumor activity of CysSs in the presence of AuNPs,^12^ an efficient antimicrobial agent against both types of bacteria.^13^ Still, however, there are only a limited number of reports studying the effect of NPs on the antimicrobial activity of AASs, including LAE.

This study focuses on the evaluation of the antibacterial activity of the d-amino acid surfactant (d-AAS) and the eco-friendly development of AAS-coated gold (Au) and silica (Si) nanoparticles (denoted as AAS-AuNPs and -SiNPs). It was found that d-LAE, d-proline dodecyl ester (d-PD), and d-alanine dodecyl ester (d-AD) showed antibacterial activity against both Gram-positive and -negative bacteria but less efficacy against Gram-negative bacteria. The antibacterial activity of d-LAE was hardly affected by its coating on AuNPs and silica nanoparticles (SiNPs). By contrast, the antibacterial activity of d*-*PD was enhanced upon its coating on AuNP. It is anticipated that the antibacterial d-AASs and their nanocomposites with NPs are promising antimicrobial agents for combating multidrug resistance in pathogens, including MRSA and Gram-negative bacteria.^8,14−16^

## 2 Results and Discussion

### 2.1 Amino Acid-Based Surfactants

**Figure 1:**
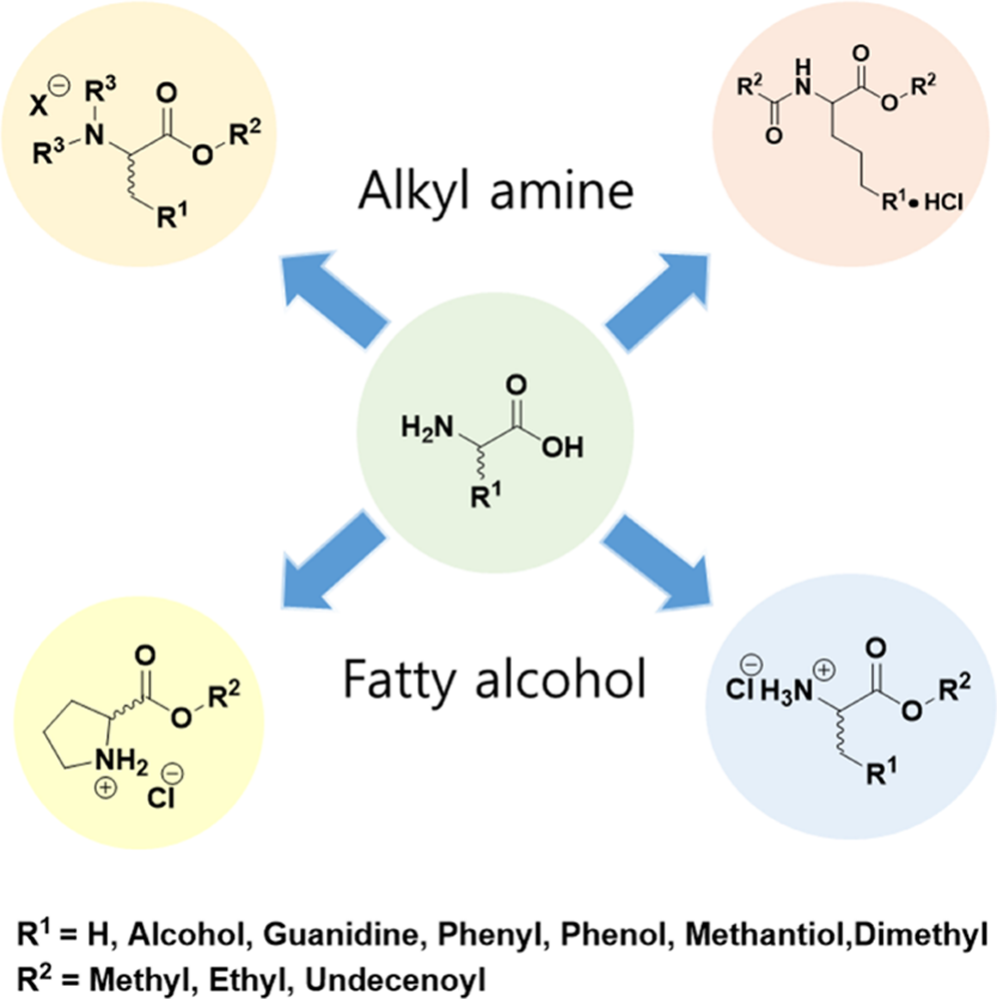
Synthesis of cationic amino acid-based surfactants.

To be suitable for industrial development, surfactants should (a) be multifunctional, (b) have low toxicity, (c) utilize renewable sources of raw materials, (d) be biodegradable, and (e) be easily synthesizable. Amino acid-based surfactants are surfactants composed of amino acids with long aliphatic chains linked through α-amino, α-COOH, or side chain groups. Surfactants synthesized from amino acids and renewable compounds have great economic and environmental potential because they are sustainable and eco-friendly materials. Common amino acids used for synthesis are glutamic acid, serine, proline, aspartic acid, glycine, arginine, alanine, leucine, etc. These compounds can be easily converted to single-chain surfactants using reactive molecules containing hydrophobic chains, such as fatty acids, fatty esters, fatty amines, and fatty alcohols (Figure 1). Hydrophobic chains can be introduced into amino acid structures via ester or amide bonds. Amino acids with reactive side chains, such as alanine, proline, or arginine, provide additional opportunities for the molecular design of single-chain surfactants. From economic and environmental viewpoints, single-chain surfactants are desirable because they can be easily used as compounds with only one amino acid in the polar head. Therefore, cationic surfactants substituted with fatty alcohols and amines for eight types of amino acids were synthesized here. The antimicrobial properties of the synthesized l*-* and d*-*amino acid surfactants were compared before they were used to coat NPs. The results are summarized in Tables 1–3.

**Table 1:** Agar Disc Diffusion for Amino Acid-Based Surfactants (Lauroyl Arginate Ethyl Ester (LAE), Dimethyl Glycinate Ethyl Ester (DGE), and Racemic (r))

diameter of inhibitory zone (mm)	d*-*LAE	l-LAE	r*-*LAE	d*-*PD^a^	l*-*PD^a^	d*-*MD^a^	d*-*AD^a^	d*-*LD^a^	d*-*WD^a^	d*-*FD^a^	DGE	gentamicin
*Vibrio cholerae*	0	0	0	14	14.5	0	13	11	0	0	0	24
*Salmonella typhimurium*	10.0	11	12	11	11	0	11	0	0	0	0	23
*Escherichia coli* (43889)	11.0	11	10.5	10	10	0	11.5	0	10.5	0	0	18.3
*E. coli* (35150)	13.0	12.5	12.3	11	11	0	11	0	0	0	0	18
*E. coli* (43890)	11.3	10.5	10.5	10	10	0	11	0	10	10	0	17.5
*Shigella boydii*	11.8	11.3	11.5	0	0	0	10	0	0	0	0	20
*Shigella flexneri*	11.5	11.6	11	10	10	0	10	0	0	0	0	18.7
*Pseudomonas aeruginosa*	11.0	10.6	11	11.8	11	0	10	0	0	0	0	19
*Enterococcus faecalis*	13.0	12.7	12.7	13.8	13	10	13	11	0	10	0	0
*Listeria monocytogenes*	15.0	14.5	14	14.5	13	10	12	11	0	0	0	23.8
*Streptococcus mutans*	16.0	15.5	15.5	16	15	10	15.5	12.5	0	0	12	17.5
*S. aureus* (MSSA)	16.5	16.5	16.3	14	13.5	10	13	11	0	0	11.5	24.3
*S. aureus* (MRSA)	15.5	15.5	15.5	13	12	10	14.5	11	0	0	11	20.3
*Bacillus subtilis*	14.3	13	13	12.5	12	0	10	10	0	0	0	24.5

a XD (X = amino acid, D = dodecyl ester).

**Table 2:** Minimum Inhibition Concentration (MIC) for Amino Acid-Based Surfactants

MIC (μg/mL)	d-LAE	l-LAE	r-LAE	d-PD^a^	l-PD^a^	d-MD^a^	d-AD^a^	d-LD^a^	d-WD^a^	d-FD^a^	DGE	gentamicin
*V. cholerae*	>200	>200	>200	25	50	>200	50	>200	>200	>200	>200	12.5
*S. typhimurium*	25	50	50	50	50	>200	50	>200	>200	>200	>200	25
*E. coli* (43889)	25	50	50	50	50	>200	100	>200	>200	>200	>200	6.25
*E. coli* (35150)	25	50	50	25	50	>200	25	>200	>200	>200	>200	12.5
*E. coli* (43890)	25	25	25	50	50	>200	25	>200	>200	>200	>200	6.25
*S. boydii*	25	50	50	>200	>200	>200	50	>200	>200	>200	>200	6.25
*S. flexneri*	50	50	50	100	100	>200	50	>200	>200	>200	>200	6.25
*P. aeruginosa*	12.5	12.5	12.5	50	100	>200	50	>200	>200	>200	>200	25
*E. faecalis*	25	25	25	25	50	>200	25	>200	>200	>200	>200	>200
*L. monocytogenes*	12.5	25	25	25	50	>200	25	>200	>200	>200	>200	3.13
*S. mutans*	12.5	12.5	12.5	12.5	12.5	>200	12.5	>200	>200	>200	>200	50
*S. aureus* (MSSA)	6.25	6.25	6.25	12.5	25	>200	12.5	>200	>200	>200	>200	1.56
*S. aureus* (MRSA)	3.13	6.25	6.25	25	25	>200	12.5	>200	>200	>200	>200	1.56
*B. subtilis*	12.5	12.5	12.5	50	50	>200	100	>200	>200	>200	>200	3.13

**Table 3:** Minimum Bactericidal Concentration (MBC) for Amino Acid-Based Surfactants

MBC (μg/mL)	d-LAE	l-LAE	r-LAE	d-PD^a^	l-PD^a^	d-MD^a^	d-AD^a^	d-LD^a^	d-WD^a^	d-FD^a^	DGE
*V. cholerae*	>200	>200	>200	50	50	>200	50	>200	>200	>200	>200
*S. typhimurium*	25	50	50	50	50	>200	50	>200	>200	>200	>200
*E. coli* (43889)	50	100	50	50	50	>200	>200	>200	>200	>200	>200
*E. coli* (35150)	50	50	50	50	25	>200	25	>200	>200	>200	>200
*E. coli* (43890)	25	25	25	50	50	>200	50	>200	>200	>200	>200
*S. boydii*	50	50	100	>200	>200	>200	50	>200	>200	>200	>200
*S. flexneri*	50	100	50	100	100	>200	50	>200	>200	>200	>200
*P. aeruginosa*	50	100	50	50	100	>200	50	>200	>200	>200	>200
*E. faecalis*	50	50	25	25	50	>200	50	>200	>200	>200	>200
*L. monocytogenes*	25	25	25	50	50	>200	50	>200	>200	>200	>200
*S. mutans*	50	50	50	12.5	12.5	>200	25	>200	>200	>200	>200
*S. aureus* (MSSA)	25	50	25	25	25	>200	50	>200	>200	>200	>200
*S. aureus* (MRSA)	12.5	50	25	25	25	>200	50	>200	>200	>200	>200
*B. subtilis*	12.5	12.5	12.5	50	50	>200	>200	>200	>200	>200	>200

We investigated the *in vitro* antimicrobial activity of the newly synthesized d*-*type amino acid derivatives toward six Gram-positive and eight Gram-negative bacteria, which are known to cause human infection. The diameter of the inhibition zone was measured to determine the antimicrobial activity using the agar diffusion method. Gentamicin was used in the same assay as a control to compare the efficacy of the tested compounds. MIC, MBC, and paper disk assay tests were performed.^17^ Both l*-* and d*-*LAEs have robust antimicrobial activity toward both Gram-negative and Gram-positive bacteria. In particular, compared to l*-*LAE, d*-*LAE has stronger antimicrobial activity toward Gram-negative bacteria.

The antimicrobial properties of d*-*amino acid-based surfactants were confirmed through the study of d*-*amino esters and amides with fatty alcohol and amines. The alanine and proline-based surfactants were shown to provide higher performance than other amino acids, implying that they should have the highest antimicrobial activity toward all tested bacteria. It is widely known that gram-negative bacteria are more resistant to antimicrobial agents than Gram-positive bacteria.^18−20^ This intrinsic resistance is attributed to the lipopolysaccharide outer membrane of the Gram-negative bacteria, which acts as an efficient permeability barrier.^21,22^ The synthesized d*-*amino acid derivatives showed promising results against *P. aeruginosa*. In addition, the results shown in Table 2 reveal that LAE was more potent than the standard antimicrobial drug gentamicin against *P. aeruginosa and S. mutans*.

### 2.2 Physicochemical Properties of Amino Acid-Based Surfactants and Gold Nanocomposite (AAS-AuNPs)

**Figure 2:**
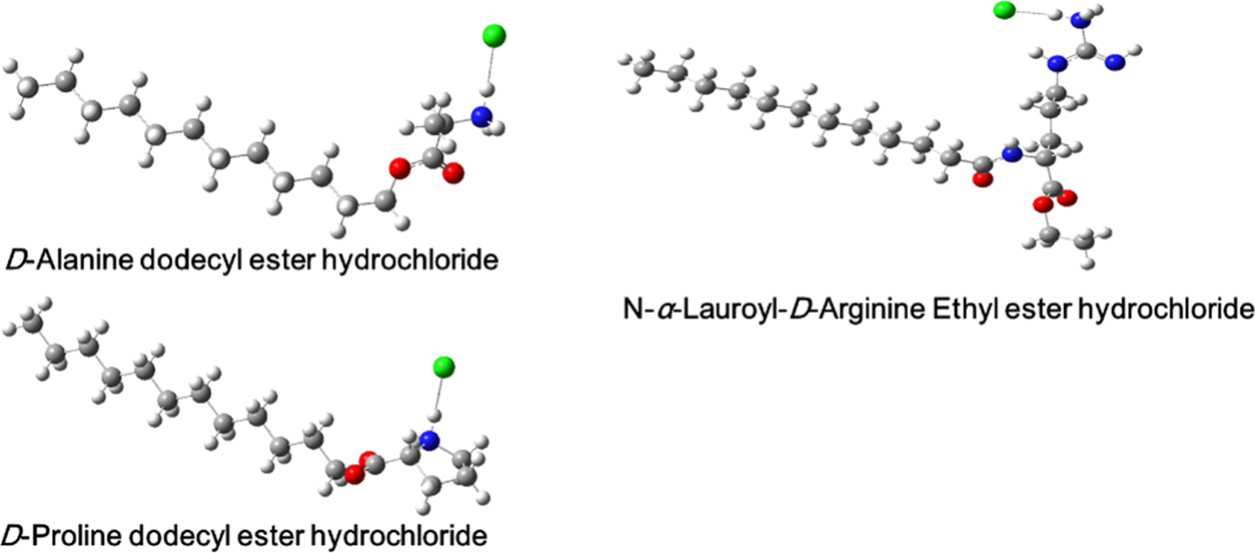
Optimized molecular structures of amino acids with dodecyl side chains, d*-*LAE, d*-*PD, and d*-*AD in water. The structures were optimized based on the Becke3–Lee–Yang–Parr (B3LYP) level with 6-311++G(d,p) basis calculations.

Although the pharmacological properties of small molecules have been widely investigated,^23−27^ studies on the chemical quantum analysis and spectroscopic and structural properties of small molecules in nanoparticles are limited. In particular, the physicochemical properties of Au nanoparticles have been analyzed through density functional theory (DFT) approaches (geometrics, HOMO–LUMO orbitals, natural binding orbitals, and morphology analysis) based on experimental data (Raman and Fourier transform infrared (FTIR) spectroscopies) of d*-*type surfactants. Information on the detailed description of the structures and properties of d*-*LAE, proline, and alanine structures is provided in Figure 2 and Table 4. To the best of our knowledge, no theoretical molecular modeling studies have been presented to discuss the spectral behavior of nanoparticles using quantum chemical DFT approaches. It is worth noting that theoretical quantum models such as DFT and advanced software programming serve as effective tools for studying the properties of multiple compounds.^28−30^

**Table 4:** Optimized Molecular Structures of d-LAE, d-PD, and d-AD in Water

property	d-LAE	d-AD	d-PD
EE + thermal free energy correction	–1690.9667	–1255.7649	–1333.1251
EE + thermal enthalpy correction	–1690.8516	–1255.6763	–1333.0337
EE + thermal energy correction	–1690.8525	–1255.6773	–1333.0346
EE + zero-point energy	–1690.8883	–1255.7027	–1333.0609
*E* (thermal), kcal/mol	410.3520	308.2840	332.1410
entropy (*S*), cal/mol-K	242.2230	186.4260	192.3990
polarizability (α), au	379.3937	265.7133	292.2987
dipole moment, Debye	13.1640	15.0786	14.3500
*E*_HOMO_ (eV)	–0.2568	–0.2620	–0.2614
*E*_LUMO_ (eV)	–0.0303	–0.0445	–0.0427
energy gap (eV) HOMO–LUMO gap (Δ_HL_)	0.2265	0.2174	0.2187
ionization potential, *I* (eV)	0.2568	0.2620	0.2614
electron affinity, *A* (eV)	0.0303	0.0445	0.0427
electronegativity, χ (eV)	0.1436	0.1532	0.1521
global hardness, η (eV)	0.1133	0.1087	0.1093
chemical potential, μ (eV)	–0.1436	–0.1532	–0.1521
global electrophilicity, ω (eV)	0.0910	0.1080	0.1058
chemical softness, *S* (eV^–1^)	8.8300	9.1979	9.1470

In this study, d*-*LAE, alanine, and proline surfactants were introduced into AuNPs to confirm their antimicrobial activity. The physicochemical properties of the synthesized nanoparticles were confirmed through FTIR and Raman spectroscopy and DFT calculations (Figure S56). All DFT calculations of the molecule were performed using the Gaussian 6.0 program package at the Becke3–Lee–Yang–Parr (B3LYP) level with a 6-311++G(d,p) basis set (Figure 2 and Table 4).

Enhanced dipole moments in drug design can enhance hydrogen-bonding and nonbonding interactions in drug–receptor complexes, which retain a vital role in increasing binding affinity. In addition, the energies of HOMO and LUMO play an important role in chemical reactivity. The HOMO and LUMO band gap is related to the physiochemical index of the molecules.^31^ Compounds with the lowest energy gap and softness may exhibit higher chemical activity and polarizability than others. Because adding electrons to the higher-positioned LUMO or removing electrons from the lower-positioned HOMO is energetically favorable for all potential reactions.

The HOMO–LUMO gap, hardness, and softness were calculated for d-LAE, d-AD, and d-PD (Table 5). As can be seen from the results in Table 5, d-AD has the lowest energy gap (0.2174 eV) and the highest softness (9.1979 eV), which can contribute to higher chemical reactivity. To experimentally confirm the above calculation results, AAS and gold nanocomposites were synthesized environmentally friendly. The synthesized AAS + AuNPs was confirmed by Raman spectroscopy, IR, TGA, SEM, TEM, and EDS. FTIR and Raman spectra show identical characteristic bands from a theoretical point of view, calculated for the geometry and vibrational modes of hydrated AAS at DFT/B3LYP levels in water solvents. These peaks confirmed a network structure between the AAS and NPs inside the nanoparticles.^32−41^

**Table 5:** Minimum Inhibition Concentration (MIC) for AAS-AuNPs

MIC (μg/mL)	1 mM AuNP	l-LAE (1 mM AuNP)	d-LAE (1 mM AuNP)	l-PD (1 mM AuNP)	d-PD (1 mM AuNP)	d-AD (1 mM AuNP)	l-PD (0.5 mM AuNP)	d-PD (0.5 mM AuNP)	d-AD (0.5 mM AuNP)	l-PD (0.5 mM AuNP)	d-PD (0.1 mM AuNP)	d-AD (0.1 mM AuNP)
*E. coli* (ATCC 25922)	>256	64	32	32	8	4	32	16	8	32	16	8

In particular, when the stability of AAS was verified through ζ-potential measurement, d-AD + AuNps was in the most stable state with a ζ-potential (54.82 mV) in the water solvent phase stability at room temperature (Figure S38). In addition, the chemical reactivity of the gold nanocomposite was confirmed through antimicrobial experiments.

### 2.3 Antimicrobial Activity of Amino Acid-Based Surfactants-Gold Nanoparticles (AAS-AuNPs)

#### 2.3.1 MIC Assay with *E. coli*

Lysogeny broth (LB) media containing increasing concentrations of different metals from AAS-AuNPs were inoculated with *E. coli*. The optical density was determined after incubating the cultures for 24 h.

AuNPs were chosen because of their ease of preparation, controllable particle size, good solubility in a wide range of buffers, surface modification, and good biocompatibility. To replace the harmful reducing agents with biocompatible and eco-friendly compounds, amino acids were used in this study. Experimental results showed that the antimicrobial activity of the AAS was enhanced when delivered with AuNPs, confirming that the AAS have antimicrobial activity. To verify that AAS-coated AuNPs have similar activity, the antimicrobial activity of AAS-coated NPs toward *E. coli* was evaluated, as described in the Methods section. l-LAE containing 1 mM AuNPs could inhibit the growth of *E. coli* at the same minimum concentration of 64 μg/mL (Table 5).

In contrast, AAS-AuNPs prepared using the equivalent of AuNPs showed inhibition at 32–4 μg/mL against *E. coli*. In addition, even when the AuNP concentration was reduced to 0.1 and 0.5 mM, inhibition was demonstrated at 8–32 μg/mL (Table 5). The results showed that the antimicrobial activity of LAE-coated AuNPs was higher than that of LAE alone. In particular, the MIC of d*-*PD was half that of l*-*PD. In addition, d-AD showed a 2-fold increase in inhibition than d-PD, confirming improved antimicrobial properties (Table 5).

### 2.4 Antimicrobial Amino Acid-Based Surfactants-Gold Nanoparticles: Modes of Action

**Figure 3:**
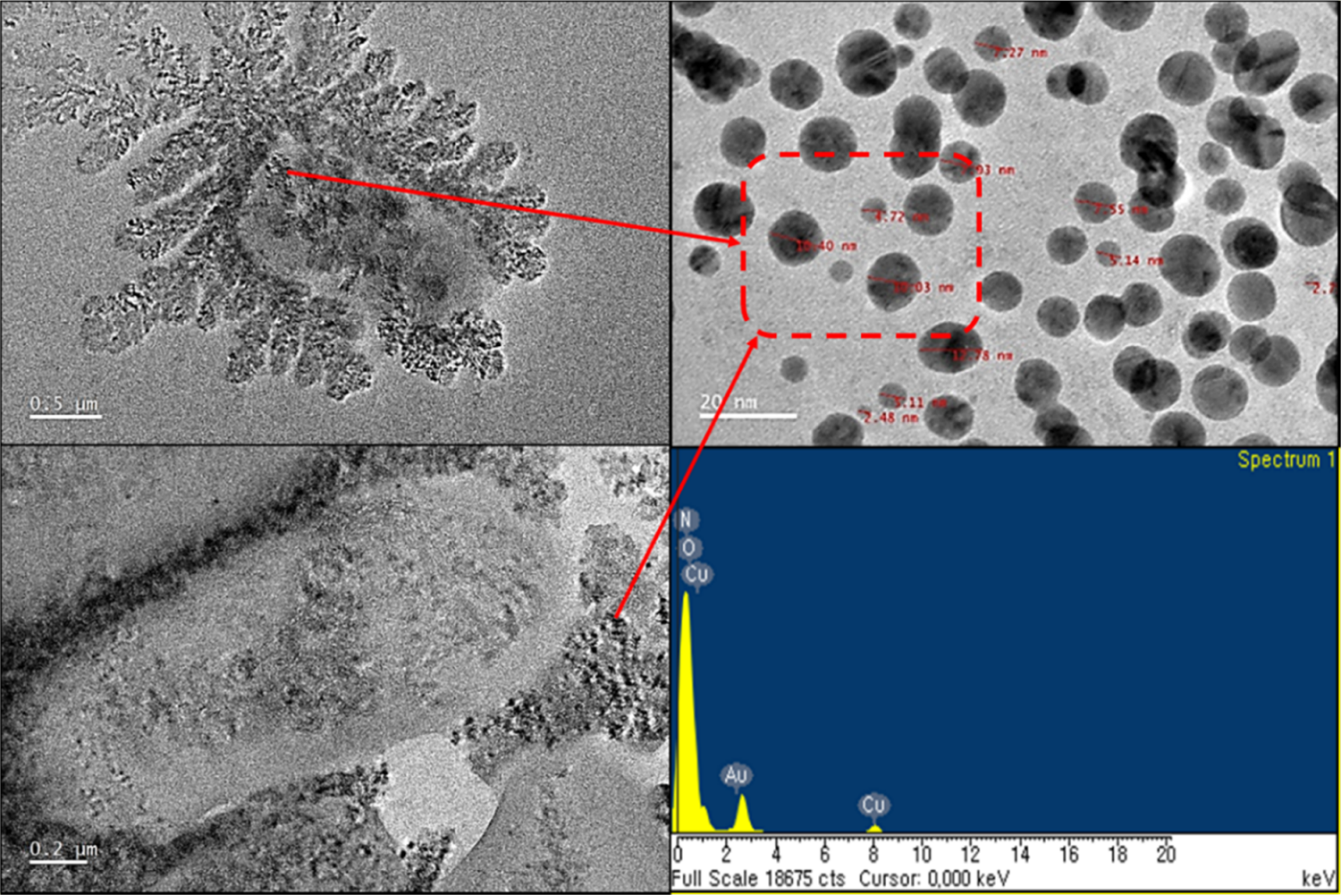
TEM and EDS analysis of *E. coli* bacteria after 24 h treatment with d-AD + AuNPs.

Based on the toroidal pore model, AAS-AuNPs adopt a well-defined secondary structure after coming in contact with the phospholipid membrane.^16^ The binding of AAS-AuNPs to the target membrane causes cell infiltration, resulting in leakage of cellular components, ultimately leading to cell destruction. In the carpet model, pore formation does not occur because AAS-AuNPs gather parallel to the bacterial membrane to cover the membrane surface like a carpet. The AAS attached to the surface trigger membrane penetrates the membrane, causing it to collapse in a detergent-like manner, subsequently forming micelles. One possible reason for the increased antimicrobial activity of AAS-AuNPs on individual components is that the AAS enables a higher drug concentration at the site of action. In addition, AAS-AuNPs can be precipitated on the membrane through interaction with lipopolysaccharides and proteins in the outer membrane of the bacteria.^42,43^ Via covalent immobilization of d-AD onto AuNPs, it significantly increased the antibacterial and antibiofilm activities against *E. coli* (Figure 3).^44,45^

According to several reports, cationic AMP is known to cause membrane depolarization and damage by binding to the negative charge of the bacterial membrane.^46^ Therefore, cationic AAS can induce depolarization of the bacterial membrane because it has similar properties to AMP.^47−49^

However, although cationic AAS induces depolarization in bacterial cell membranes, this does not imply high antimicrobial activity. In a similar study, the correlation between bacterial membrane depolarization and antimicrobial activity was compared using cationic amphiphilic Gramicidin S and polymyxin, and it was reported that depolarization and bacterial cell lethality did not correlate well.^50^

In addition, the polar head of the cationic surfactant (cetyltrimethylammonium chloride, CTAC) interacts with AuNPs at concentrations below CMC and concentrations above CMC; CTACs form micelles and interact with surrounding AuNPs. Therefore, according to the CTAC and AuNPs models, since micelles of cationic AAS interact with AuNPs and surround them, the antibacterial activity of cationic AAS-AuNPs seems to be determined by the antibacterial activity of AAS rather than the effect of AuNPs.^51^

### 2.5 Antimicrobial Activity of Amino Acid-Based Surfactants-Silica Nanoparticles (AAS-SiNPs)

The growth inhibitions of *E. coli* (ATCC 25922), *S. aureus* (ATCC 6538), *Candida albicans* (ATCC 10231), and *S. mutans* (ATCC 25175) were tested using the film adhesion method to confirm the antimicrobial effect of the composite. As shown in Table 6, d*-*LAE-SiNP and d*-*LAE-SiNP-Si^a^ exhibited reduction rates of 95% or more against all evaluated strains. Despite the low content of d*-*LAE-SiNPs in the d*-*LAE-SiNP-Si^a^, it showed practical antimicrobial ability toward Gram-negative and Gram-positive bacteria. In the case of *C. albicans* fungus (ATCC 10231), d*-*LAE-SiNPs showed excellent antimicrobial activity, but the d*-*LAE-SiNP-Si^a^ complex showed a reduction rate of only ≤9%.^52,53^

**Table 6:** *In Vitro* Antimicrobial Activity of AAS-SiNPs against *E. coli* (ATCC 25922), *S. aureus* (ATCC 6538), *C. albicans* (ATCC 10231), and *S. mutans* (ATCC 25175); the Growth Inhibition Rate of Microbials Is the Reduction Rate

bacteria	sample	reduction rate (%)
*E. coli* (ATCC 25922)	blank	NA
	d*-*LAE	99.9
	d*-*LAE-SiNP	99.9
	d*-*LAE-SiNP- Si^a^	99.3
*S. aureus* (ATCC 6538)	blank	NA
	d*-*LAE	99.9
	d*-*LAE-SiNP	99.9
	d*-*LAE-SiNP-Si^a^	97.0
*C. albicans* (ATCC 10231)	blank	NA
	d*-*LAE	99.9
	d*-*LAE-SiNP	99.9
	d*-*LAE-SiNP-Si^a^	9.0
*S. mutans* (ATCC 25175)	blank	NA
	d*-*LAE-SiNP-Si^a^	95.7

**Figure 4:**
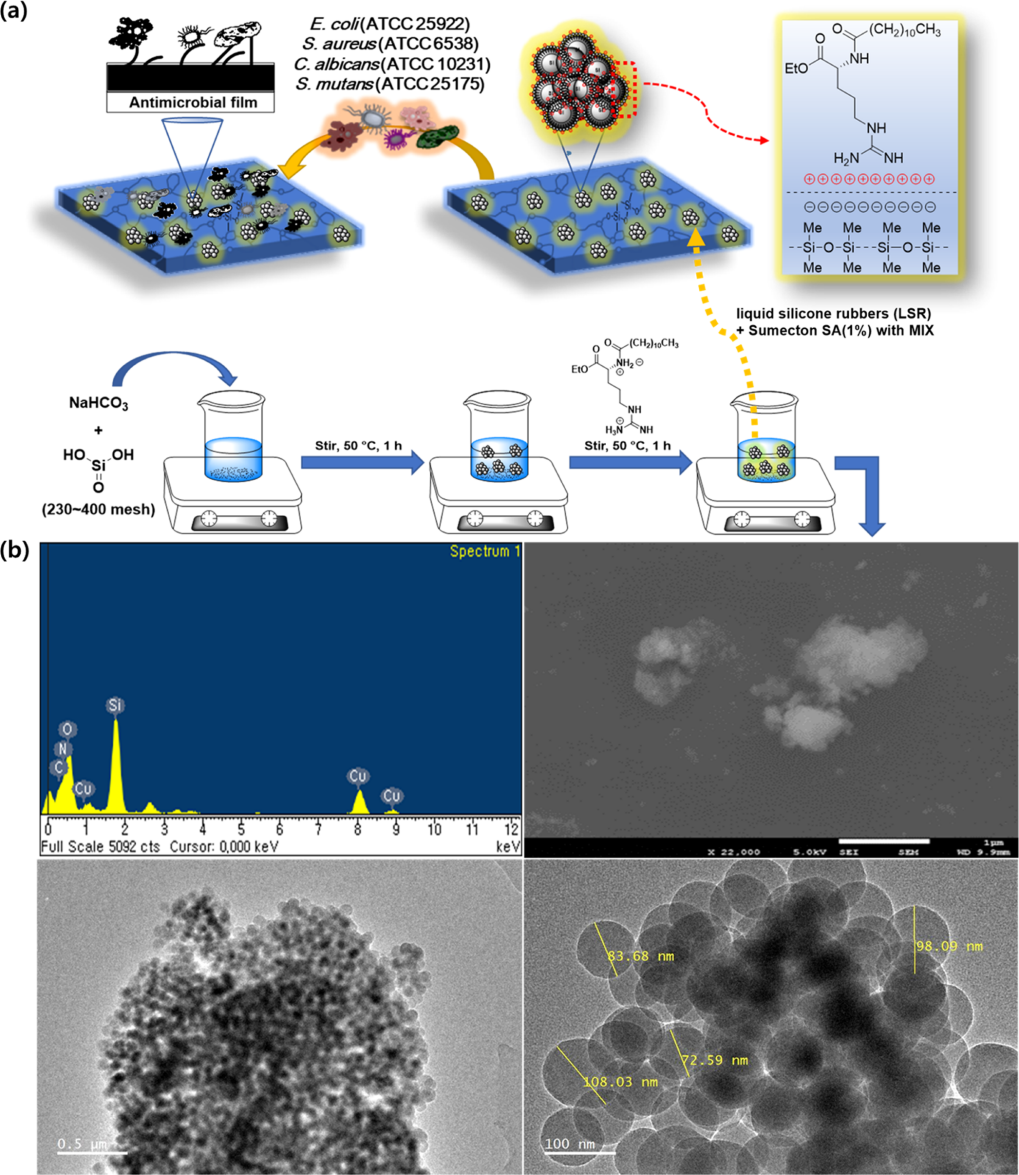
Possible mechanisms and analysis of d-LAE-SiNP-Si^a^ against multidrug-resistant (MDR) bacteria. (a) Synthesis scheme for functionalized d-LAE-SiNP-Si^a^. Schematic illustration of functionalized SiNP-Si^a^ with various functional groups. (b) Characterization of SiNPs: Scanning electron microscopy images of SiNP; up-right scale bars: 1 μm. Energy-dispersive X-ray spectroscopy (EDS) elemental spectra of SiNPs: up-left. High-resolution transmission electron microscopy (TEM) images of as-prepared SiNPs, and cross-sectional TEM images of SiNPs. Scale bars: down-left, 0.5 μm; down-right, 100 nm. The EDS spectrum pattern showed that SiNPs still contained the AAS after the reaction.

A possible mechanism for the formation of AAS-SiNPs is proposed as follows. First, the size of the SiNPs is ∼100 nm. Therefore, the contact area between SiNPs and AAS was large enough to induce more interactions. Second, the pH value of the surface modification process was adjusted to 8 to form many hydroxyl groups on the silica surface. Therefore, the surface charge of the SiNPs was negative. When the cationic surfactant AAS was added to the system, the ionic interaction between the positive and negative charges on the surface of SiNPs arranged the organic chains of AAS around the nanosilica, as shown in Figure 4a. Finally, AAS reduced the surface energy of SiNPs by reacting with the hydroxyl groups on the silica surface to graft the surfactant to the silica surface. After the organic chain of AAS was grafted onto the surface of SiNPs, the three-dimensional structure between NPs became more significant, leading to more SiNPs being monodispersed. This increased the stability and improved the dispersion state of the SiNPs.^54−58^

### 2.6 Cytotoxicity Study of Amino Acid-Based Surfactants-Gold, Silica Nanoparticles (AAS-Au, SiNPs)

**Figure 5:**
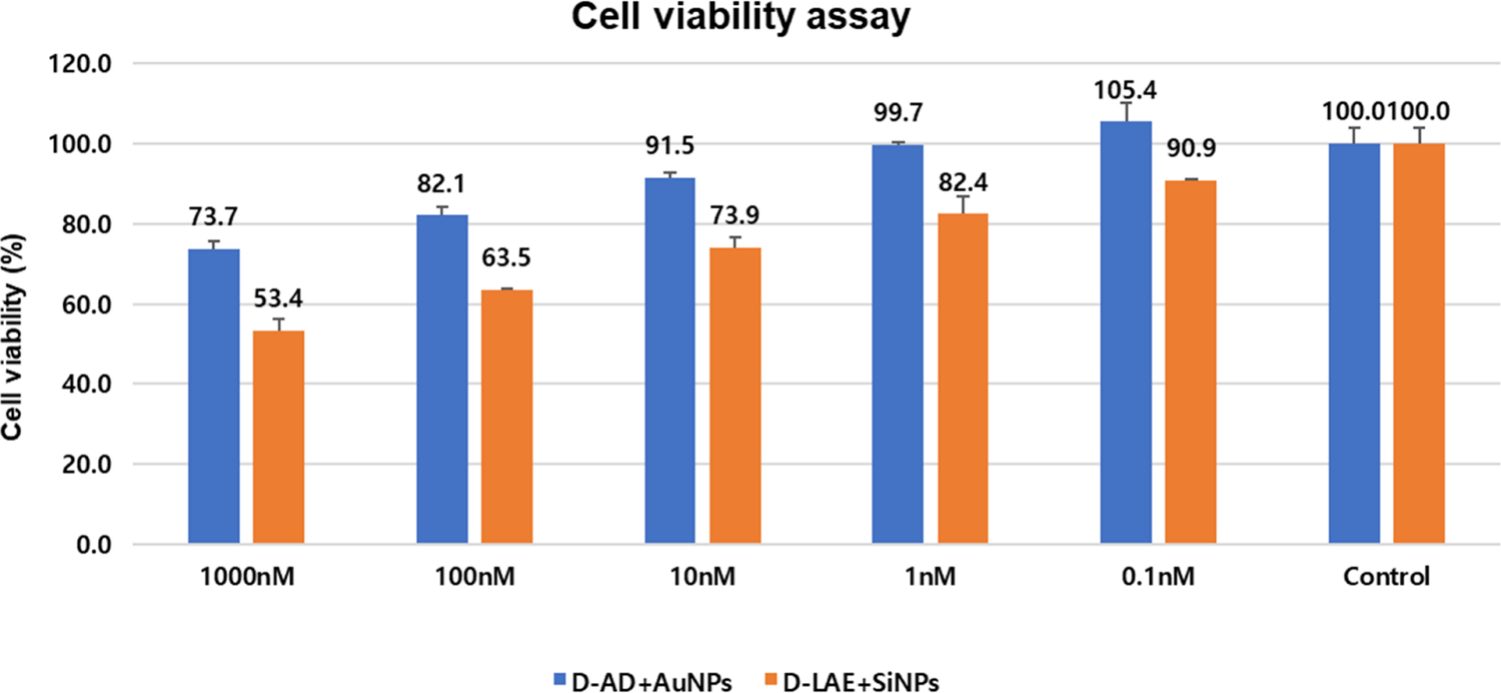
d-AD+AuNPs and d-LAE + SiNP MTT assay.

As shown in Figure 5, the cytotoxicity of environmentally synthesized d-AD + AuNPs and d-LAE + SiNPs was investigated in human embryonic kidney 293T cells. Cell viability (100% cell viability) expressed as a percentage of AAS+NPs at various concentrations ranging from 1000 to 0.1 nM and untreated control HEK293T cells was investigated using the cell count kit 8 assay kit. As a result of analyzing the analysis results for 48 h, d-AD + AuNPs showed a survival rate of 91.5% at a concentration of 10 nM, and d-LAE + SiNPs showed a survival rate of 90.9% at a concentration of 0.1 nM. However, a negative slope was found with increasing concentrations of cytotoxicity in high concentrations of nanoparticle complexes. Therefore, our present study showed that the synthesized d-AD + AuNPs had low toxicity to HEK293T cells up to 10 nM and d-LAE + SiNPs up to 0.1 nM.

## 3 Conclusions

In conclusion, we synthesized various d-AASs and evaluated their antimicrobial activity. We found that d-LAE, d-PD, and d-AD showed antibacterial activity against Gram-positive and -negative bacteria but less efficacy against Gram-negative bacteria. The antibacterial activity of d-LAE against Gram-negative bacteria was higher than that of d-PD and d-AD. The antibacterial activity of d-LAE remained unaffected even when it was coated on AuNP and SiNP, whereas that of d-PD and d-AD was enhanced upon its coating on AuNP. The antibacterial activity against Gram-negative bacteria was higher for d-LAE than l-LAE but similar between d-PD and l-PD and l-LAE and l-PD. The antibacterial activity of both l-LAE and l-PD was only slightly enhanced upon the coating on AuNP. Thus, the effect of amino acid chirality on the antibacterial activity of AASs and their nanocomposites with NPs depends on the type of amino acids in the AASs. We propose that the antibacterial d-AASs and their nanocomposites with AuNPs and SiNPs are potential antimicrobial agents against MDR pathogens.

## 4 Experimental Section

### 4.1 Vibrational Spectral Analysis

FTIR spectra of the synthetic compounds were recorded at room temperature in the 5000–500 cm^–1^ range using a Bruker IFS 66V model spectrometer equipped with a mercury–cadmium–telluride detector, KBr beam splitter, and Globar arc source. Fourier transform (FT)-Raman spectra of 1-BN were recorded on a computer-interfaced Bruker IFS 66V model interferometer equipped with an FRA-106 FT-Raman accessory. FT-Raman spectra were measured in the 5000–50 cm^–1^ Stokes region using a Nd:YAG laser continuously operated at 200 mW power with 1064 nm excitation. The reported wavenumbers were expected to demonstrate ±1 cm^–1^ accuracy. The computed vibrational wavenumbers and atomic displacements corresponding to the different normal modes were used for the identification of vibrational modes. The vibrational modes were assigned on the basis of potential energy distribution analysis using the VEDA4 program.^26,36^

### 4.2 Preparation and Characterization of AuNPs, SiNPs, and Silicone Complexes

#### 4.2.1 Method for Preparing AAS-AuNP

Herein, AuNPs were synthesized using a surfactant-free emulsion method, unlike the conventional AuNP synthesis process. Au^3+^ ions and natural extracts in HAuCl_4_ reduced Au^3+^ ions through a redox reaction. d*-*type LAE, proline, and alanine derivatives were added at different concentrations (0.1, 0.5, 1 mM) to form Au composite NPs.^29,39^ After preparing AAS-AuNPs, an MIC test was performed to evaluate whether the Au nanoparticle could affect the antimicrobial activity.

#### 4.2.2 Method for Preparing d*-*LAE-SiNP

Silica gel 60 (230–400 mesh; 1 equiv) is added to distilled water, and NaHCO_3_ (2.1 equiv) is added to that, followed by stirring at 50 °C for 1 h. After the reaction, the synthesized d*-*LAE was added at a concentration of 0.4% of the silica solution and then stirred for about 1 h. After cooling to room temperature slowly, citric acid is added dropwise to adjust the pH to 4–5. The completion of the reaction is confirmed using FTIR, SEM, TEM, and EDS.

#### 4.2.3 Method for Preparing d*-*LAE-SiNP-Si^a^ Complex

The silicone resin used in this study was ELASTOSIL LR 3002/35 as A/B to prepare silicone Rubber A (silicone polymer + Pt catalyst) and silicone Rubber B (silicone polymer, crosslinker, inhibitor) liquid silicone rubber (mixing ratio 1:1). d*-*LAE-SiNP water solution (4%) and part A and part B were mixed at room temperature using a typical hand mixer. A portion of the molded mold and the specimen (mold temperature of 140 °C, 4 h) were taken. The fabricated sample was dried at room temperature for 12 h.

### 4.3 Theoretical Calculations

All DFT calculations of the molecule were performed using the Gaussian 6.0 program package^14,23^ at the Becke3–Lee–Yang–Parr (B3LYP) level with a 6-311++G(d,p) basis set. The structural parameters were computed in the gas and liquid phases using a polarizable continuum model method. The calculated wavenumbers were scaled using a constant scaling factor to correct the overestimations arising from negative aspects, such as the basis set truncation effect, neglection of electron correlations, and anharmonicity characters of the vibrational modes at 0.9673. The optimized geometrical parameters, fundamental vibrational frequencies, IR intensity, Raman activity, atomic charges, dipole moment, and other thermodynamical parameters were calculated^59^ using the GAUSSIAN 16W package.

### 4.4 Agar Disk Diffusion Method

To determine the antimicrobial susceptibility of AASs, the agar disk diffusion method was used. Briefly, filter paper disks of 8 mm in diameter were prepared and sterilized. Using sterile forceps, the disks were aseptically placed over nutrient agar plates seeded with the respective test microorganisms. The plates were incubated overnight at 37 °C. The diameter of the inhibition zone around the disk was recorded as bacterial growth inhibition. The diameters of the inhibition zones (in mm) were measured, and the experiments were performed three times.^60^

### 4.5 Minimal Inhibitory Concentration (MIC) and Minimal Bactericidal Concentration (MBC)

After the disk diffusion test, MIC and MBC were determined to quantify the antimicrobial activity of the formulations. Various concentrations of AAS, AAS-AuNP, AAS–SiNP, and AAS–SiNP-Si^a^ (3.1–100 μg/mL) were inoculated with bacterial culture in 96-well plates. MIC was determined after 24 h of incubation at 37 °C by observing the visible turbidity and measuring the optical density of these culture broths at OD 600 nm. MIC is defined as the lowest concentration of an antimicrobial agent that inhibits the growth of microorganisms. In contrast, MBC is defined as the lowest concentration of an antimicrobial agent that kills 99.9% of the initial bacterial population. The MBC values were determined by removing 100 μL of bacterial suspension from the culture, demonstrating no visible growth in the MIC experiment, and inoculating in agar plates. The plates were incubated at 37 °C for 24 h to determine MBC.

### 4.6 Cytotoxicity Assay

A cytotoxicity test was performed to confirm the cytotoxicity of d-AD + AuNPs and d-LAE + SiNPs with human embryonic kidney 293T cells. After diluting test substances prepared with 500 mM DMSO stock to the final treatment concentration, human embryonic kidney 293T cells (4 × 10^4^ cells/well) were treated in a 96-well plate for cytotoxicity test in an incubator at 37 °C, CO_2_ and incubated for 48 h. d-AD + AuNPs, d-LAE + SiNPs were treated with 5 concentrations (1000, 100, 10, 1, and 0.1 nM), and the final DMSO % was 0.7%. After 48 h of incubation, 10 μL of the cell count kit 8 (WST-8/CCK8, Dojindo Lab, CK04) reagent was added, and after 1 h, absorbance was measured at 460 nm using a microplate reader.

## Supporting Information Available

Characterization of products (copies of ^1^H, ^13^C, HRMS, SEM, TEM, ζ, TGA, and computation data) (PDF)
