# Supplementary material for: Investigation of d-Amino Acid-Based Surfactants and Nanocomposites with Gold and Silica Nanoparticles as against Multidrug-Resistant Bacteria Agents
Source: ACS Omega. 2022 Dec 8;7(50):46146–55. doi: 10.1021/acsomega.2c04220 (PMC9773340; doi:10.1021/acsomega.2c04220)

Supporting Information

Investigation of *D*-Amino Acid-Based Surfactants and Nanocomposites with Gold and Silica Nanoparticles as against multidrug-resistant(MDR) bacteria agents

Jae Ho Shim[*a‡], Sung Duk Gwak[b‡], Byung Kook Ahn[a], Hogyu Han[b], Yeonsun Hong[c], Ok Sarah Shin*[*d]*

[a]*Department of Anatomy, Korea University College of Medicine, Seoul 02842, Korea*
[b]*Department of Chemistry, Korea University, Seoul 02841, Korea*
[c]*Department of Microbiology and Immunology, David H. Smith Center for Vaccine Biology and Immunology, University of Rochester, Rochester, NY 14642, USA*
[d]*BK21 Graduate Program, Department of Biomedical Sciences, College of Medicine, Korea University Guro Hospital, Seoul, Korea*
** E-mail: shimjh3000@korea.ac.kr, oshin@korea.ac.kr*
‡ Jae Ho Shim and Seong Deok Kwak contributed equally to this paper

Table of Contents

1. Instruments and reagents……………………………………………………………… S-2
2. Synthesis of products; general procedure…………………………………………… S-2
3 Compound Characterization Data…………………………………………………… S-4
4. References……………………………………………………………………………. S-6
5. Copy of NMR and MASS Spectra………………………………………………….... S-7
6. Copy of TGA Data…………………………………………………………………… S-23
7. Copy of SEM Data…………………………………………………………………… S-26
8. Copy of Zeta Potential Analysis Data………………………………………………… S-29
9. Computational Results of DFT Calculations for all Calculated Structures………. S-32
10. Calculated and Experimental FTIR DATA……………………………………… S-42
11. Copy of XRD Data………………………………………………………………… S-49

1. Instruments and reagents

IR spectrum was recorded using Bomen MB-104 spectrophotometer, and optical rotation was measured using Rudolph Research Autopol III polarimeter. ^{1}H NMR and ^{13}C NMR spectrum were obtained using JeolJNM-LA400 (400 MHz) using DMSO-d_6 and TMS internal standards. HRMS spectra were obtained using a JEOL JMS-AX505WA mass spectrometer. The reagents used in this study were products such as Aldrich, TCI chemical, and if necessary, purified or dried by a known method. Merck's silica gel 60 (230-400mesh) was used as a stationary phase for column chromatography. FESEM imaging was performed on samples dispensed on ultrathin carbon lm coated gold grids (Ted Pella Inc., USA). zeta potential, and polydispersity were assessed using a Zeta-potential & Particle size Analyzer ELSZ-2000 series (Otsuka Electronics, JAPEN). FT-IR spectrum for functional group detection was analyzed by PerkinElmer Spectrum Two (USA). Confocal Raman spectra were acquired on a Horiba LabRAM HR 3Evolution micro-Raman spectrometer (Germany). HR-TEM images were obtained on a JEOL 2100F microscope operating at 200 kV accelerating voltage EDS was performed in STEM mode on a JEOL 2100F instrument (UK). TGA experiments were performed by a TA Instruments Thermogravimetric Analyzer (TGA Q500, USA).

2. Synthesis of products; general procedure

(1) Synthesis of monoalkylated products.

To a stirred solution of *D*-amino acid (5 g, 56.1 mmol) in toluene (100 mL) was added dodecanol 2 (9.42 g, 50,5 mrnol) in one lot, followed by *p*TSA(Toluene-4-sulfonic acid monohydrate, 11.75 g, 61.7mmol). After addition, the temperature of the reaction mixture was slowly raised to reflux temperature, the water was separated, and the reaction mixture was monitored by TLC. The reaction mixture was concentrated under vacuum, the obtained residue was taken in ethyl acetate (200 mL) and washed with aqueous 5%Na_2CO_3 (3x50 mL) followed by brine solution. The organic layer was dried over NaS and concentrated under vacuum to afford crude dodecyl compounds as a liquid. A stirred solution of Compound (5 g, 17,54mmol) in ethyl acetate/hexane/MeOH (10:10:1 mL) was cooled to 0°C. The reaction mixture was stirr with 1*N* HCl for 60 minutes at 50°C, and the reaction mixture was monitored by TLC. The reaction mixture was concentrated under vacuum, and the obtained residue was Hushed with ethyl acetate (3x52 mL) followed by hexane (5x50 mL) to afford wet compounds (5.5 g) as a semi solid. The semi solid was taken in ethyl acetate/hexane (10:10 mL) and heated to reflux, and stirred at reflux for 30 minutes. The reaction mixture was slowly cooled to RT and then to 0°C. The obtained solid was filtered under nitrogen and dried under vacuum to afford ester salt as a white hygroscopic solid

(2) Synthesis of dialkylated products.

A mixture of the amino acid (12.1 mmol), *p*TSA (2.30 g, 12.1 mmol) and alcohol (C2~C12, 14.5 mmol) in

toluene (100 mL) was heated for 48 h instead of 24 h using a Dean–Stark apparatus. The crude products were purified by column chromatography on silica gel with EtOAC/Hexane (1:2) to afford the corresponding esters as oils. The esters were then converted to the *N*,*N*-diethyl derivatives using EtBr/K_2CO_3 in a sealed tube at 90°C. The QUATS (5b–8b) were then synthesised by heating the corresponding *N*,*N*-diethyl derivatives with excess MeI in a sealed tube at 90°C for 18 h. The solvent was removed under vacuo and on addition of diethyl ether the desired quaternary ammonium compound precipitated out.

(3) Synthesis of N-*α*-alkyl-*D*-Arginine Ethyl ester hydrochloride products.

To 100 mL of two necks round bottom flask, 21 g of *D*-Arginine 2HCl and 20 g of $CaCl_2$ were added, 200 mL of EtOH was added, stirred for 10 minutes, 10 mL of H_2SO_4 was added, and refluxed at 100°C for 8 hours. After cooling to room temperature, the mixture was concentrated under reduced pressure to remove EtOH, and 200 mL of distilled water was added to the reaction precipitate to disperse while stirring. 52 g of $NaHCO_3$ was slowly added, then 200 mL of ethyl acetate (EA) was added and stirred, and 22 mL of alkyl chloride was slowly added, followed by stirring at room temperature for 3 hours. While stirring the reaction mixture, adjust the pH between 4 and 5 with concentrated hydrochloric acid, add 200 mL of EA, and separate the organic layer, remove moisture from the organic layer with $MgSO_4$, filter, and then concentrated under reduced pressure to obtain a white powder product.

3. Compound Characterization Data

1. N-α-Lauroyl-*D*-Arginine Ethyl ester hydrochloride(*D*-LAE) (1a) (Yield 95%)[1]

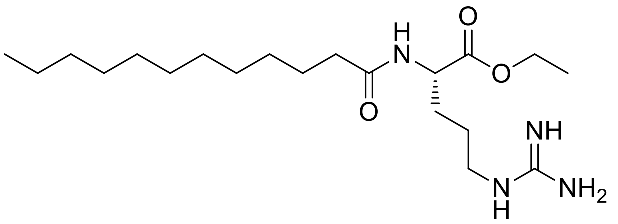

^{1}H NMR(DMSO-d_6), δ (ppm): 8.22~8.23 [d, 1H, (-NH-CH-COO)], 7.82~85 [t, 1H, (-NH-C(=NH)-NH_2)], 7.03~36 [br s, 3H, (-NH-C(=NH)-NH_2)], 4.10~16 [m, 1H, (-NH-CHCOO-)], 4.0~07 [m, 2H, (-OCH_2-CH_3)], 3.05-3.07 [m, 2H, (CH_2-NH-C(=NH)-NH_2)]], 2.06~10 [t, 2H, (-CH_2CONH-)], 2.03 [s, 1H, (-NH-C(=NH)-NH_2)],1.54-71 [m, 2H, (CO-CH_2, CH_2-CH_2-)] 1.42-1.48 [m, 4H, (CH_2-CH_2-)] 1.20 [br s, 18H, (9CH_2, alkyl chain)], 1.12-16 [t, 3H, (OCH_2-CH_3)], 0.80~84 [t, 3H, (CH_3 alkyl chain)]; ^{13}C NMR(DMSO-d_6), δ (ppm): 177.1, 176.6, 161.5, 64.9, 56.2, 39.5, 35.8, 33.57, 33.53, 33.37, 33.2, 33.1, 32.45, 29.8, 29.7, 26.6, 18.6, 18.5; HRMS (FAB+) for $C_{20}H_{40}N_4O_3$ $[M+H]^+$ Calcd: 384.3100, Found: 385.3175.

2. *D*-Proline dodecyl ester hydrochloride(*D*-PD) (1b) (Yield: 87%)[2]

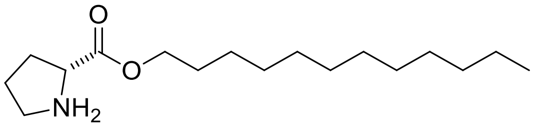

^{1}H NMR(DMSO-d_6), δ (ppm): 4.28~4.32[m, 1H, (-CHCOO-CH_2)], 4.08~4.14[m, 2H, (-CH_2-CH_2-)], 3.14~3.20[m, 2H, (-CH_2-CH_2-)], 1.90~2.23[m, 2H, (-CH_2-CH_2-)], 1.85~1.89[m, 2H, (-CH2-CH2-)], 1.54~1.59[m, 2H, (-CH_2-CH_2-)], 1.21[br s, 18H, (9CH_2, alkyl chain)], 0.80~0.84[t, 3H, (CH_3 alkyl chain)]; ^{13}C NMR(DMSO-d_6), δ (ppm): 169.33, 66.35, 58.92, 45.62, 31.83, 29.58, 29.55, 29.52, 29.25, 28.4, 28.26, 25.72, 23.54, 22.63, 14.47; HRMS (FAB+) for $C_{17}H_{34}NO_2$ $[M+H]^+$ Calcd: 284.2590, Found: 284.2585.

3. *D*-Alanine dodecyl ester hydrochloride(*D*-AD) (1c) (Yield: 93%)[3]

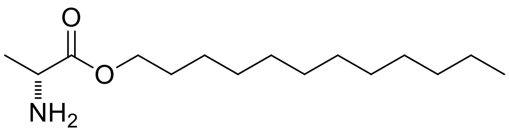

^{1}H NMR(DMSO-d_6), δ (ppm): 8.60[brs, 3H, (-CHCOO-NH_3^+)], 4.07~4.14[m, 2H, (-CHCOO-CH_2-)], 3.97~4.02[m, 1H, (-CHCOO-CH-)], 1.54~58[m, 2H, (-CH_2-CH_2-)], 1.38~1.39[d, 3H, (-CH_3-CH-)], 1.21 [brs, 18H, (9CH2, alkyl chain)], 0.80~84 [t, 3H, (CH_3 alkyl chain)]; ^{13}C NMR(DMSO-d_6), δ (ppm): 170.54, 66.03, 61.23, 48.36, 33.08, 31.83, 29.64, 29.57, 29.54, 29.51, 29.24, 29.14, 28.49, 25.70, 22.62, 16.24, 14.47; HRMS (FAB+) for $C_{15}H_{31}NO_2$ $[M+H]^+$ Calcd: 257.2355, Found: 258.2434.

4. *D*-Phenylalanine dodecyl ester hydrochloride(*D*-FD) (1d) (Yield: 89%)[3]

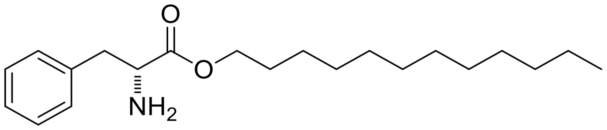

^{1}H NMR(DMSO-d_6), δ (ppm):8.75 [brs, 3H, (-CHCOO-NH_3^+)], 7.20~31 [m, 5H, (-Phenyl-CH)], 4.16~19 [m, 1H, (-CHCOO-CH-)], 3.96~99 [m, 2H, (-CHCOO-CH_2-)], 2.99~3.24 [dd, 2H, (-CH_2-CH_2-)], 1.32~40 [m, 2H, (-CH_2-CH_2-)], 1.09~26 [m, 18H, (9CH_2, alkyl chain)], 0.80~84 [t, 3H, (CH_3 alkyl chain)]; ^{13}C NMR(DMSO-d_6), δ (ppm): 169.57, 135.39, 129.87, 129.02, 127.67, 65.99, 53.74, 36.55, 31.83, 29.57, 29.54, 29.51, 29.40, 29.25, 29.12, 28.32, 25.65, 22.63, 14.48; HRMS (FAB+) for $C_{21}H_{35}NO_2$ $[M+H]^+$ Calcd: 333.2668, Found: 334.2744.

5. *D*-Leucine dodecyl ester hydrochloride(*D*-LD) (1e) (Yield: 90%)[4]

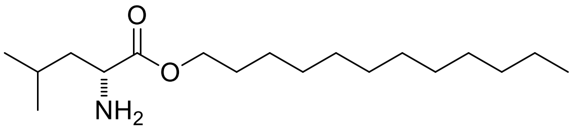

^{1}H NMR(DMSO-d_6), δ (ppm): 8.63[brs, 3H, (-CHCOO-NH_3^+)], 4.06~14[m, 2H, (-CHCOO-CH2-)], 3.84~3.87[m, 1H, (-CHCOO-CH-)], 1.72~75[m, 1H, (-CH_2-CH-CH_2-)], 1.59~65[m, 1H, (-CH-CH_2-CH-)], 1.53~58[m, 2H, (-CH_2-CH_2-)], 1.21[brs, 18H, (9CH_2, alkyl chain)], 0.85-87 [dd, 6H, (-CH_3-CH-CH_3-)], 0.80~84 [t, 3H, (CH_3 alkyl chain)]; ^{13}C NMR(DMSO-d_6), δ (ppm): 170.42, 66.03, 51.05, 31.83, 29.56, 29.45, 29.23, 29.08, 28.43, 25.75, 24.35, 22.77, 22.62, 22.42, 14.46; HRMS (FAB+) for $C_{18}H_{37}NO_2$ $[M+H]^+$ Calcd: 299.2824, Found: 300.2894.

6. *D*-Methionine dodecyl ester hydrochloride(*D*-MD) (1f) (Yield: 83%)[5]

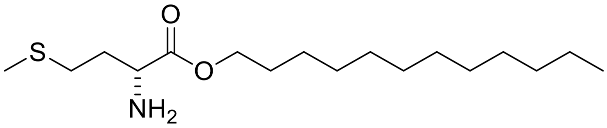

^{1}H NMR(DMSO-d_6), δ (ppm): 8.71[brs, 3H, (-CHCOO-NH_3^+)], 4.13~4.16[m, 2H, (-CHCOO-CH_2-)], 4.07~4.09[m, 1H, (-CHCOO-CH-)], 2.48~2.65[m, 2H, (-S-CH_2-)], 2.06~2.09[m, 2H, (-S-CH_2-CH_2-)], 2.02[s, 3H, (-S-CH_3-)], 1.57~1.62[m, 2H, (-CH_2-CH_2-)], 1.24[brs, 18H, (9CH_2, alkyl chain)], 0.80~0.84[t, 3H, (CH_3 alkyl chain)]; ^{13}C NMR(DMSO-d_6), δ (ppm): 169.74, 66.20, 51.36, 31.83, 29.97, 29.58, 29.54, 29.49, 29.47, 29.24, 29.13, 28.96, 28.44, 25.79, 22.63, 14.77, 14.48; HRMS (FAB+) for $C_{17}H_{35}NO_2S$ $[M+H]^+$ Calcd: 317.2389, Found: 318.2465.

7. *D*-Tyrosine dodecyl ester hydrochloride(*D*-YD) (1g) (Yield: 84%)[6]

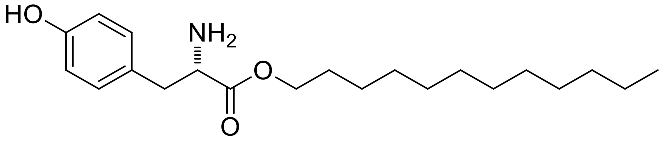

^{1}H NMR(DMSO-d_6), δ (ppm): 9.42 [br s, 1H, (-Phenyl-OH)], 8.78[br s, 3H, (-CHCOO-NH_3^+)], 6.96~98 [d, 2H,

(-Phenyl-CH)], 6.67~69 [d, 2H, (-Phenyl-CH)], 4.07~09 [m, 1H, (-CHCOO-CH-)], 3.98~4.01 [m, 2H, (-CHCOO-CH_2-)], 2.89~3.09 [dd, 2H, (-CH_2-CH_2-)], 1.42~45 [m, 2H, (-CH_2-CH_2-)], 1.21 [br s, 18H, (9CH_2, alkyl chain)], 0.80~84 [t, 3H, (CH_3 alkyl chain)]; ^{13}C NMR(DMSO-d_6), δ (ppm): 169.66, 157.27, 130.84, 124.92, 115.88, 65.99, 53.97, 35.76, 31.84, 29.59, 29.54, 29.43, 29.26, 29.16, 28.40, 25.72, 22.64, 14.49; HRMS (FAB+) for $C_{21}H_{35}NO_3$ $[M+H]^+$ Calcd: 349.2617, Found: 350.2694.

8. N, N-Dimethylglycine ethyl ester hydrochloride(MGE) (1h) (Yield: 78%)[7]

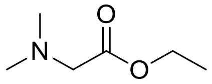

^{1}H NMR(DMSO-d_6), δ (ppm): 10.97 [brs, 1H, (-CHCOO-NH^+-$(CH_3)_2$)], 4.15~20 [m, 2H, (-CHCOO-CH_2-)], 2.80 [brs, 6H, (-CHCOO-NH^+-$(CH_3)_2$)], 2.79 [s, 2H, (-CH_2-CH_2-)], 0.80~84 [t, 3H, (CH_3 alkyl chain)]; ^{13}C NMR(DMSO-d_6), δ (ppm): 166.27, 62.20, 56.41, 56.02, 43.41, 14.43; HRMS (FAB+) for $C_6H_{13}NO_2$ $[M+H]^+$ Calcd: 131.0946, Found: 132.1025.

4. References

1. Ma, Q.; Davidson, P. M; Zhong, Q. Properties and potential food applications of lauric arginate as a cationic antimicrobial. *Int. J. Food. Microbiol.* **2020**, *315*, 108417.

2. Joondan, N.; Caumul, P.; Jhaumeer-Laulloo, S. Investigation of the physicochemical and biological properties of proline-based surfactants in single and mixed surfactant systems. *Journal of Surfactants and Detergents.* **2017**, *20*, 103–115

3. Baczko, K.**;** Larpenta, C.; Lesot, P. New amino acid-based anionic surfactants and their use as enantiodiscriminating lyotropic liquid crystalline NMR solvents. *Tetrahedron: Asymmetry* **2004**, *15*, 971–982

4. Zheng, L.; Zhao, Z.; Yang, Y.; Li, Y.; Wang, C. Novel skin permeation enhancers based on amino acid ester ionic liquid: Design and permeation mechanism. *International Journal of Pharmaceutics* **2020**, *576*, 119031

5. Desmarchelier, A.; Raynal, M.; Brocorens, P.; Vanthuyned, N.; Bouteiller, L. Revisiting the assembly of amino ester-based benzene-1,3,5-tricarboxamides: chiral rods in solution. *Chemical Communications* **2015**, *51*, 7397-7400

6. Joondan, N.; Jhaumeer-Laulloo, S.; Caumul, P. A study of the antibacterial activity of l-Phenylalanine and l-Tyrosine esters in relation to their CMCs and their interactions with 1,2-dipalmitoyl-sn-glycero-3-phosphocholine, DPPC as model membrane. *Microbiological Research* **2014**, *169*, 675–685

7. Kadyrov, R. Hydrogenolysis of Amide Acetals and Iminium Esters. *ChemCatChem* **2018**, *10*, 170-172

5. Copy of NMR and MASS Spectra

1. N-*α*-Lauroyl-*D*-Arginine Ethyl ester hydrochloride (1a)

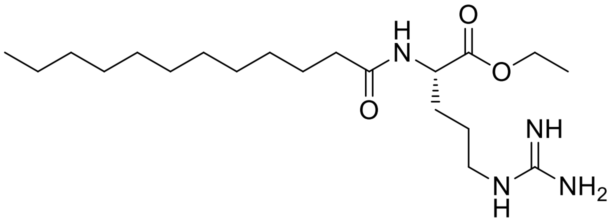

Figure S1. [1]H nmr

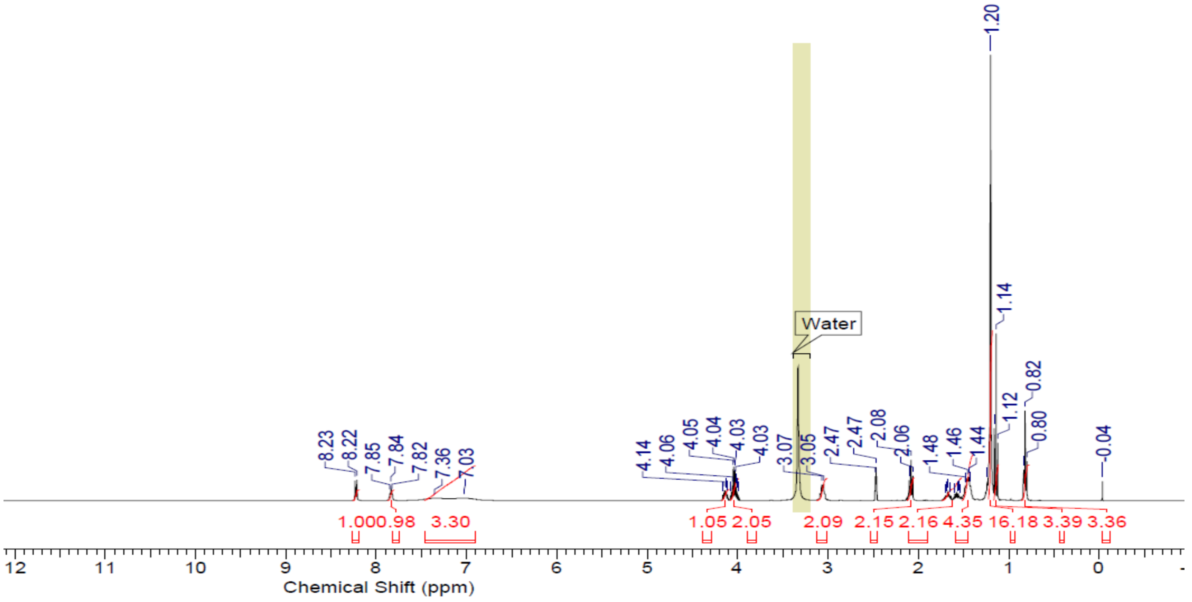

Figure S2. [13]C nmr

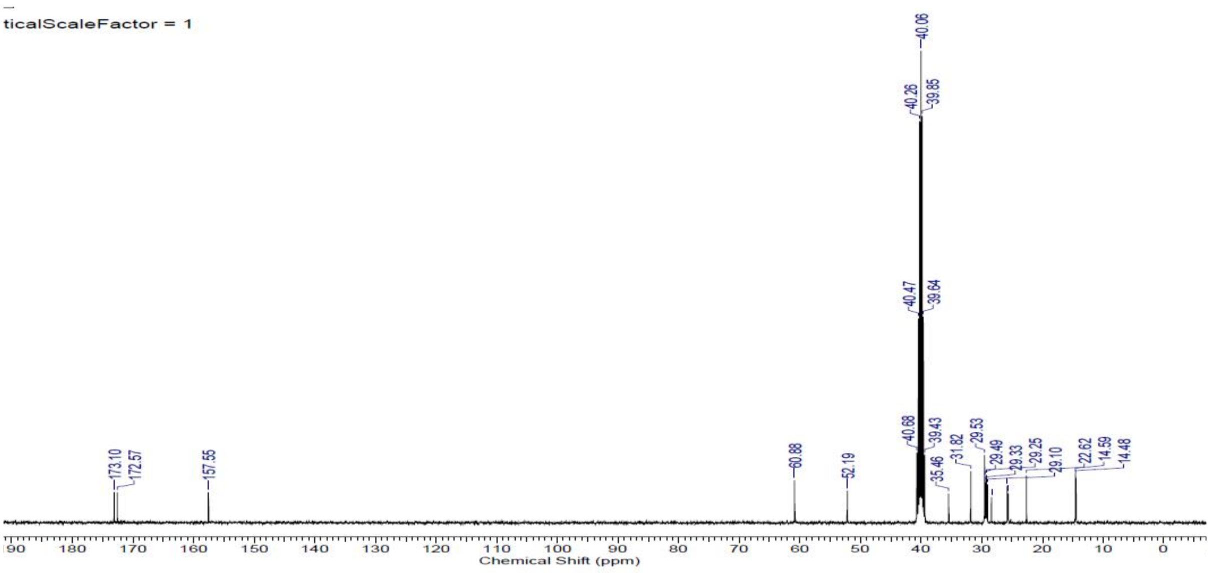

Figure S3. HRMS(FAB+)

Note : m−NBA
Inlet : Direct Ion Mode : FAB+
RT : 0.10 min Scan# : (3,5)
Elements : C 100/0, H 100/0, N 5/0, O 10/0
Mass Tolerance : 1000ppm, 5mmu if m/z < 5, 10mmu if m/z > 10
Unsaturation (U.S.) : −0.5 − 10.0

	Observed m/z	Int%	Err[ppm / mmu]	U.S.	Composition
1	385.3175	100.00	−7.9 / −3.0	7.0	C23 H39 N5
2			−11.4 / −4.4	6.5	C25 H41 N2 O
3			+21.2 / +8.2	7.0	C24 H39 N3 O
4			+17.8 / +6.8	6.5	C26 H41 O2
5			−1.0 / −0.4	2.5	C20 H41 N4 O3
6			−4.4 / −1.7	2.0	C22 H43 N O4

2. *D*-Proline dodecyl ester hydrochloride (1b)

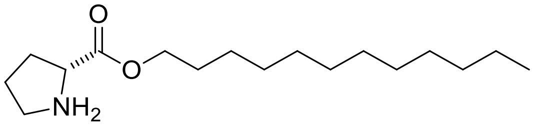

Figure S4. [1]H nmr

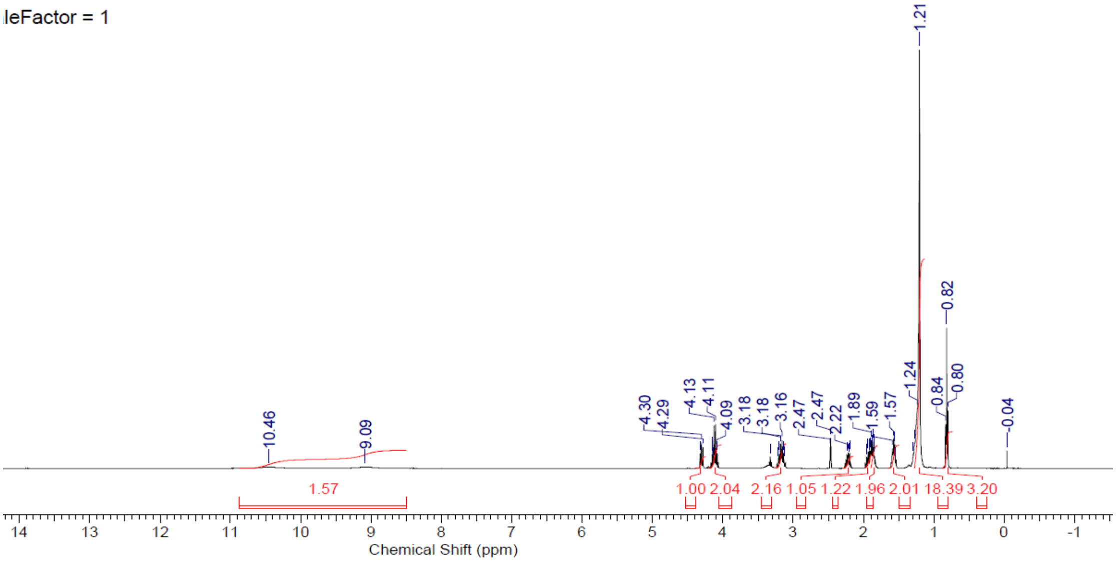

Figure S5. [13]C nmr

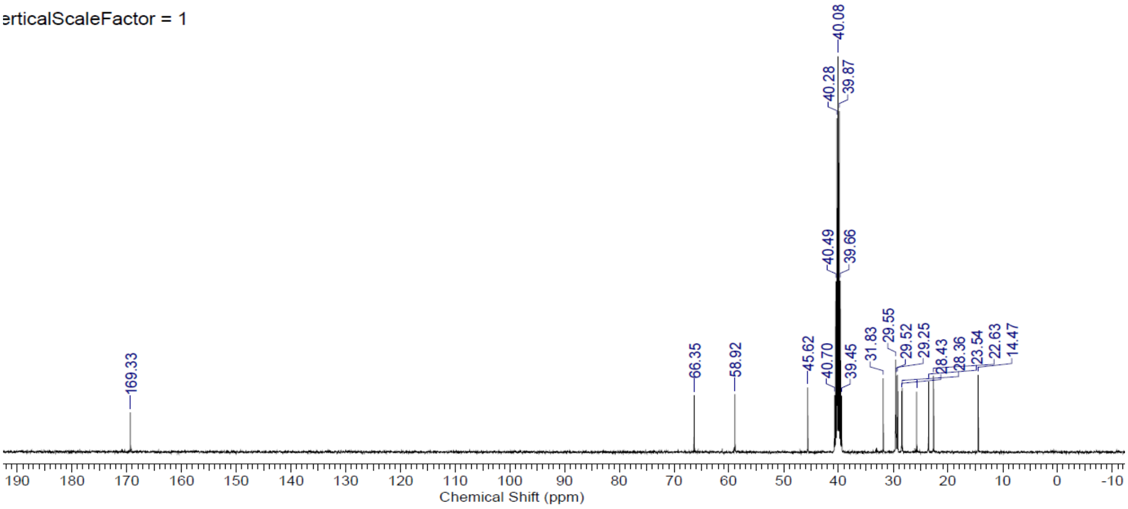

Figure S6. HRMS(FAB+)

Note : m-NBA
Inlet : Direct Ion Mode : FAB+
RT : 0.09 min Scan# : (3,34)
Elements : C 100/0, H 100/0, N 3/0, O 10/0
Mass Tolerance : 1000ppm, 5mmu if m/z < 5, 10mmu if m/z > 10
Unsaturation (U.S.) : -3.0 - 10.0

	Observed m/z	Int%	Err[ppm / mmu]	U.S.	Composition
1	284.2585	100.00	+28.5 / +8.1	6.0	C21 H32
2			-1.6 / -0.5	1.5	C17 H34 N O2
3			-31.7 / -9.0	-3.0	C13 H36 N2 O4
4			+12.6 / +3.6	-2.5	C12 H34 N3 O4
5			+7.8 / +2.2	-3.0	C14 H36 O5

3. *D*-Alanine dodecyl ester hydrochloride (1c)

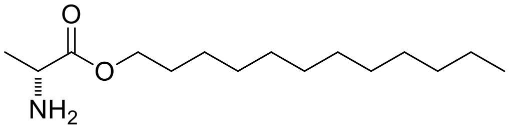

Figure S7. [1]H nmr

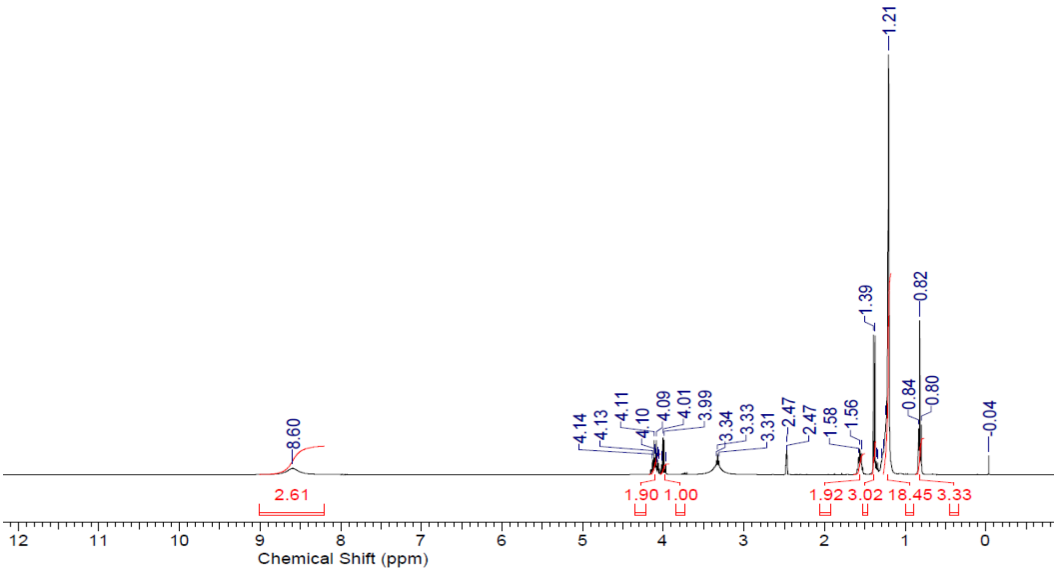

Figure S8. [13]C nmr

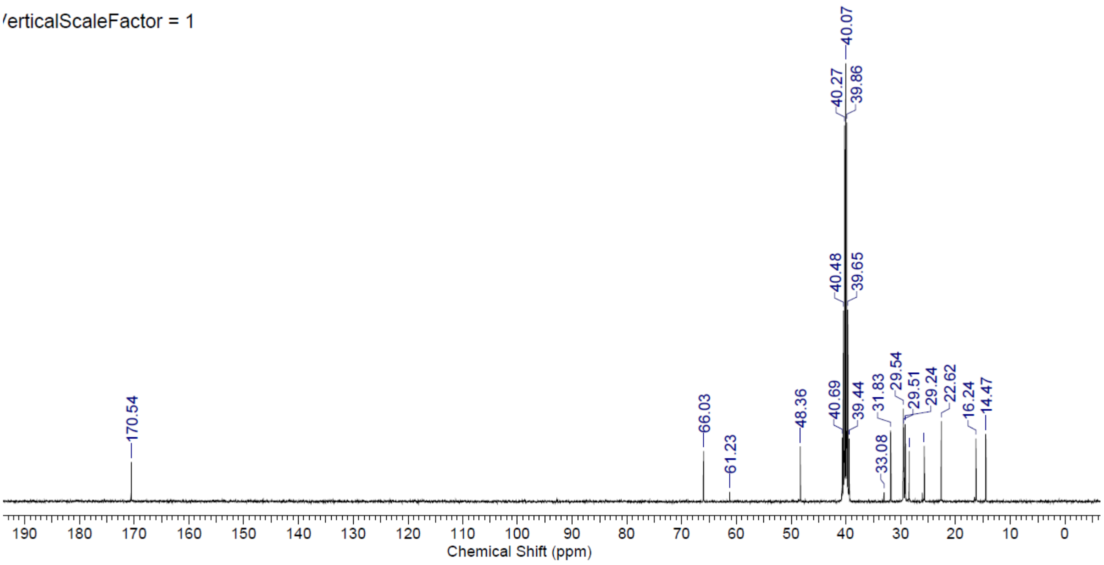

Figure S9. HRMS(FAB+)

Note : Glycerol
Inlet : Direct Ion Mode : FAB+
RT : 2.78 min Scan# : (64,73)
Elements : C 100/0, H 100/0, N 3/0, O 10/0
Mass Tolerance : 1000ppm, 5mmu if m/z < 5, 10mmu if m/z > 10
Unsaturation (U.S.) : −3.0 – 10.0

	Observed m/z	Int%	Err[ppm / mmu]	U.S.	Composition
1	258.2434	100.00	+33.5 / +8.6	5.0	C19 H30
2			+0.4 / +0.1	0.5	C15 H32 N O2

4. *D*-Phenylalanine dodecyl ester hydrochloride (1d)

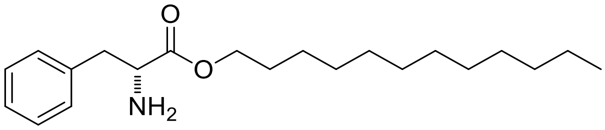

Figure S10. ^{1}H nmr

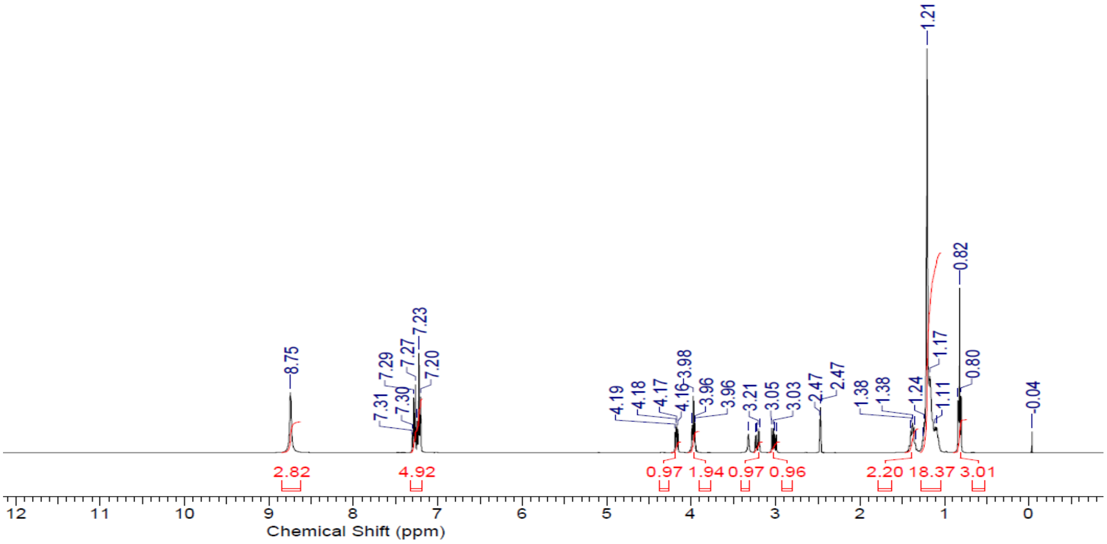

Figure S11. ^{13}C nmr

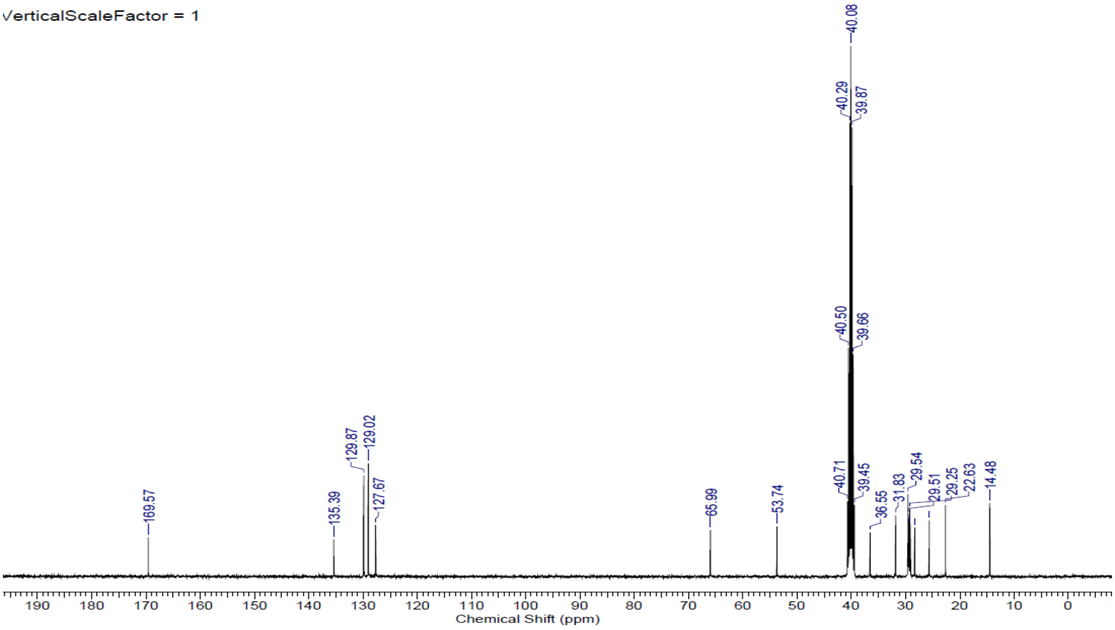

Figure S12. HRMS(FAB+)

Note : Glycerol
Inlet : Direct   Ion Mode : FAB+
RT : 1.29 min   Scan# : (31,33)
Elements : C 100/0, H 100/0, N 3/0, O 10/0
Mass Tolerance   : 1000ppm, 5mmu if m/z < 5, 10mmu if m/z > 10
Unsaturation (U.S.) : -0.5 - 10.0

	Observed m/z	Int%	Err[ppm / mmu]	U.S.	Composition
1	334.2744	100.00	+25.0 / +8.3	9.0	C25 H34
2			-0.6 / -0.2	4.5	C21 H36 N O2
3			-26.2 / -8.8	0.0	C17 H38 N2 O4
4			+11.4 / +3.8	0.5	C16 H36 N3 O4
5			+7.4 / +2.5	0.0	C18 H38 O5

5. *D*-Leucine dodecyl ester hydrochloride (1e)

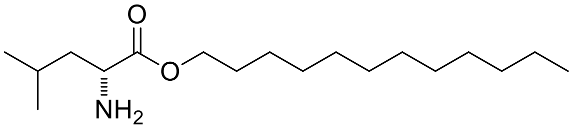

Figure S13. ^{1}H nmr

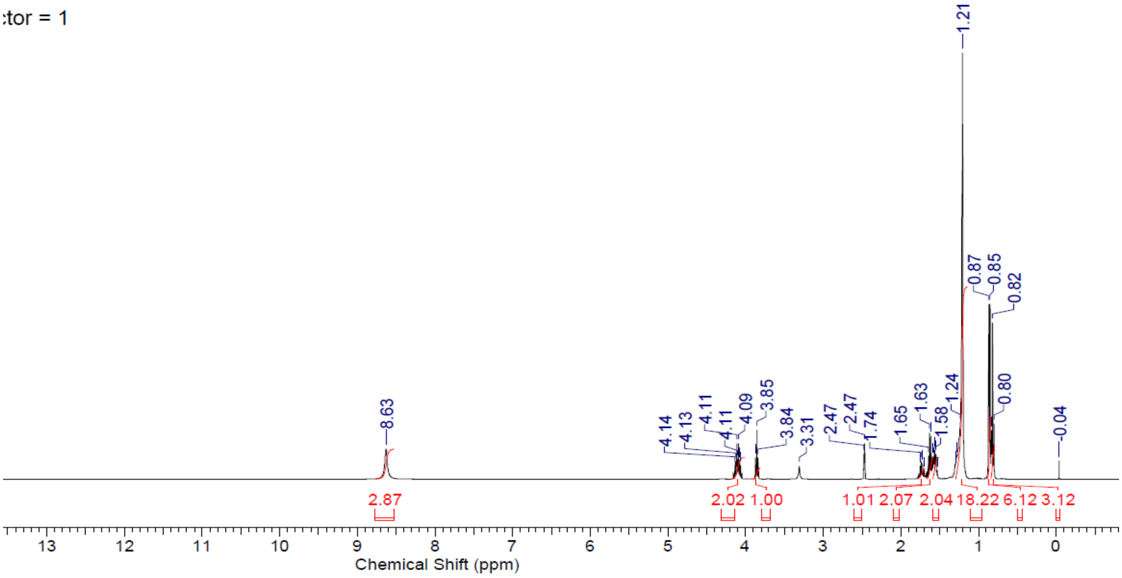

Figure S14. ^{13}C nmr

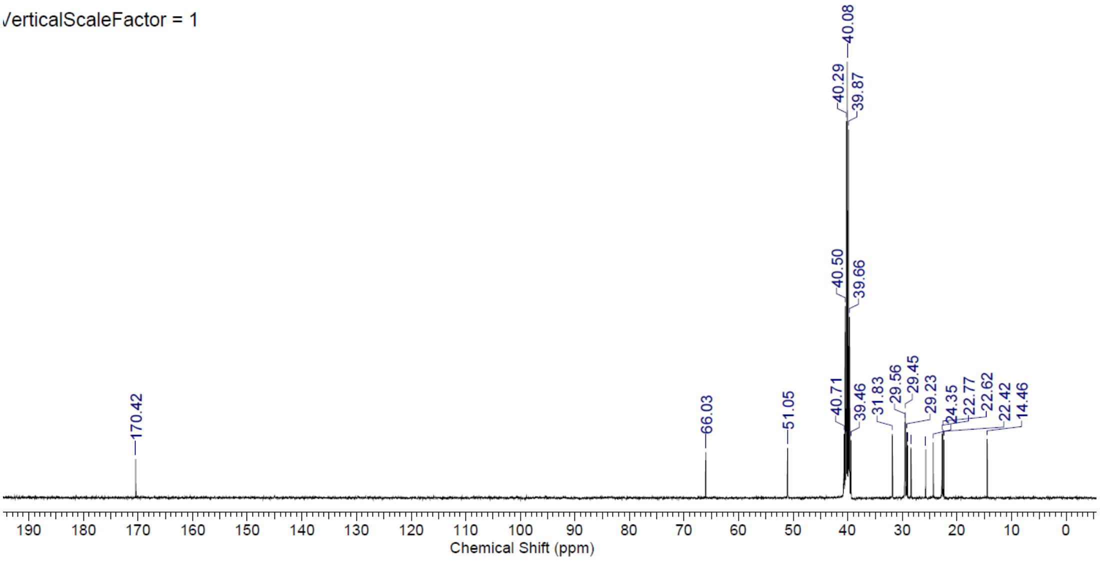

Figure S15. HRMS(FAB+)

Note : m-NBA
Inlet : Direct Ion Mode : FAB+
RT : 1.59 min Scan# : 38
Elements : C 100/0, H 100/0, N 3/0, O 10/0
Mass Tolerance : 1000ppm, 5mmu if m/z < 5, 10mmu if m/z > 10
Unsaturation (U.S.) : -3.0 - 10.0

	Observed m/z	Int%	Err[ppm / mmu]	U.S.	Composition
1	300.2894	100.00	+25.6 / +7.7	5.0	C22 H36
2			-2.8 / -0.9	0.5	C18 H38 N O2

6. *D*-Methionine dodecyl ester hydrochloride (1f)

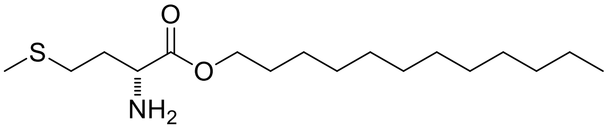

Figure S16. ^{1}H nmr

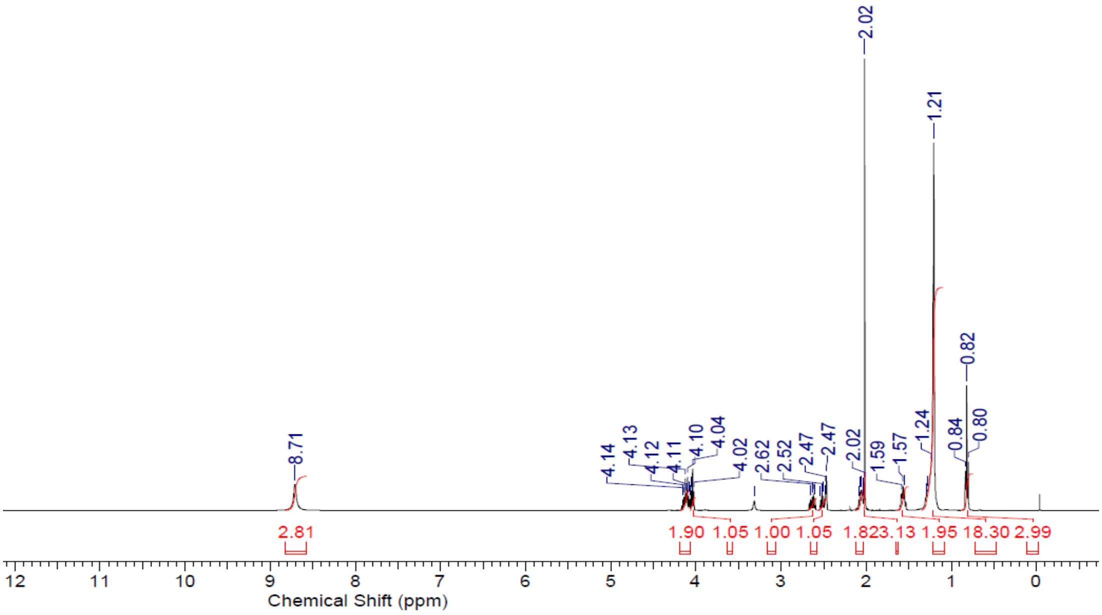

Figure S17. ^{13}C nmr

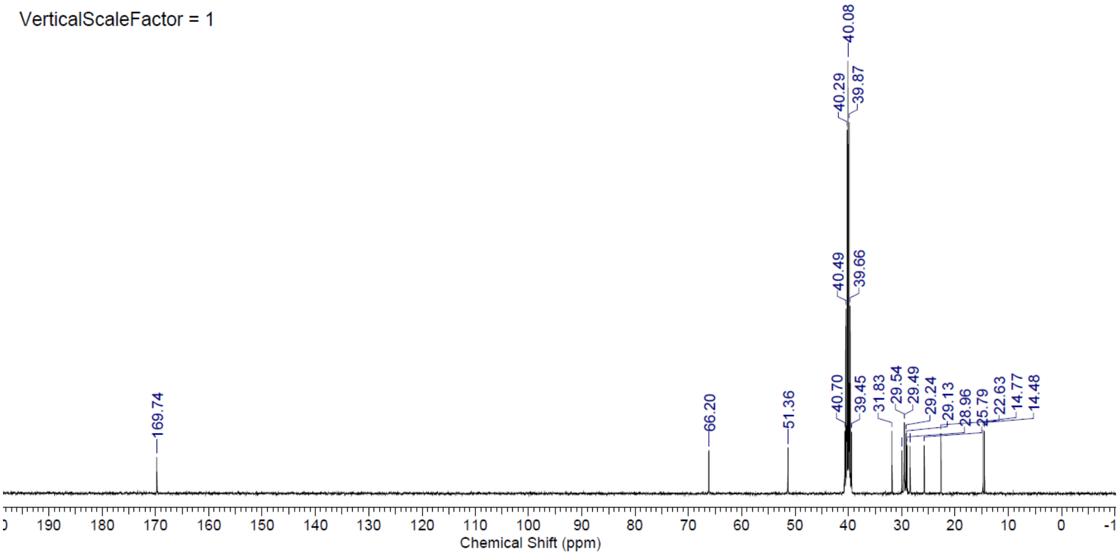

Figure S18. HRMS(FAB+)

Note : m-NBA
Inlet : Direct Ion Mode : FAB+
RT : 0.39 min Scan# : (10,20)
Elements : C 100/0, H 100/0, N 2/0, O 10/0, S 2/0
Mass Tolerance : 1000ppm, 5mmu if m/z < 5, 10mmu if m/z > 10
Unsaturation (U.S.) : -3.0 - 10.0

	Observed m/z	Int%	Err[ppm / mmu]	U.S.	Composition
1	318.2465	100.00	-29.5 / -9.4	5.0	C21 H34 O2
2			+10.0 / +3.2	5.5	C20 H32 N O2
3			-16.8 / -5.4	1.0	C16 H34 N2 O4
4			+18.5 / +5.9	1.0	C17 H34 O5
5			+31.1 / +9.9	-3.0	C12 H34 N2 O7
6			+26.3 / +8.4	6.0	C21 H34 S
7			-0.6 / -0.2	1.5	C17 H36 N O2 S
8			-27.4 / -8.7	-3.0	C13 H38 N2 O4 S
9			+7.9 / +2.5	-3.0	C14 H38 O5 S
10			+15.7 / +5.0	2.0	C18 H38 S2
11			-11.1 / -3.5	-2.5	C14 H40 N O2 S2
12			+28.4 / +9.0	-2.0	C13 H38 N2 O2 S2

7. *D*-Tyrosine dodecyl ester hydrochloride (1g)

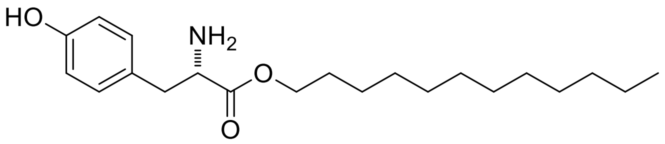

Figure S19. [1]H nmr

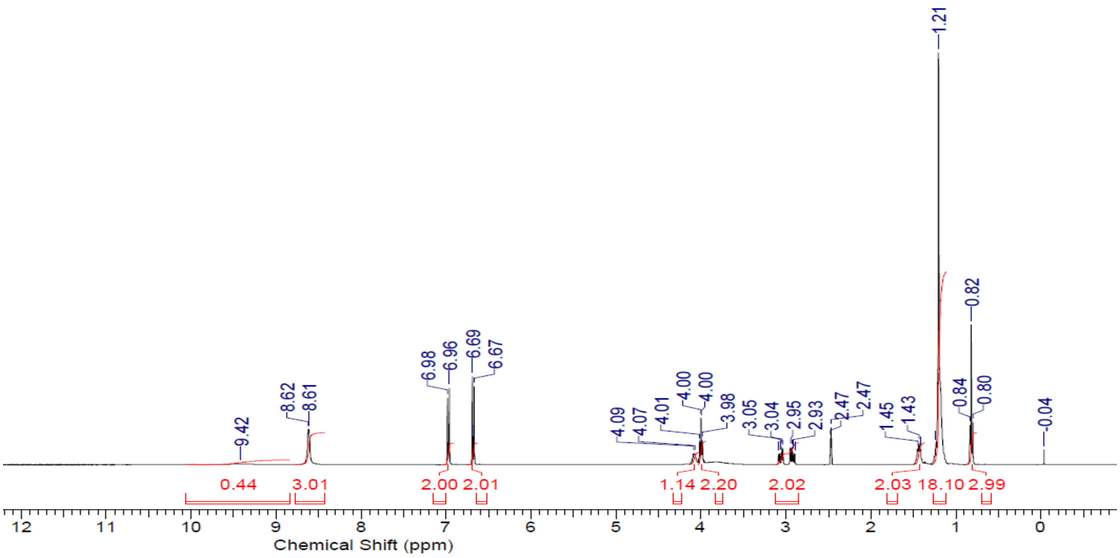

Figure S20. [13]C nmr

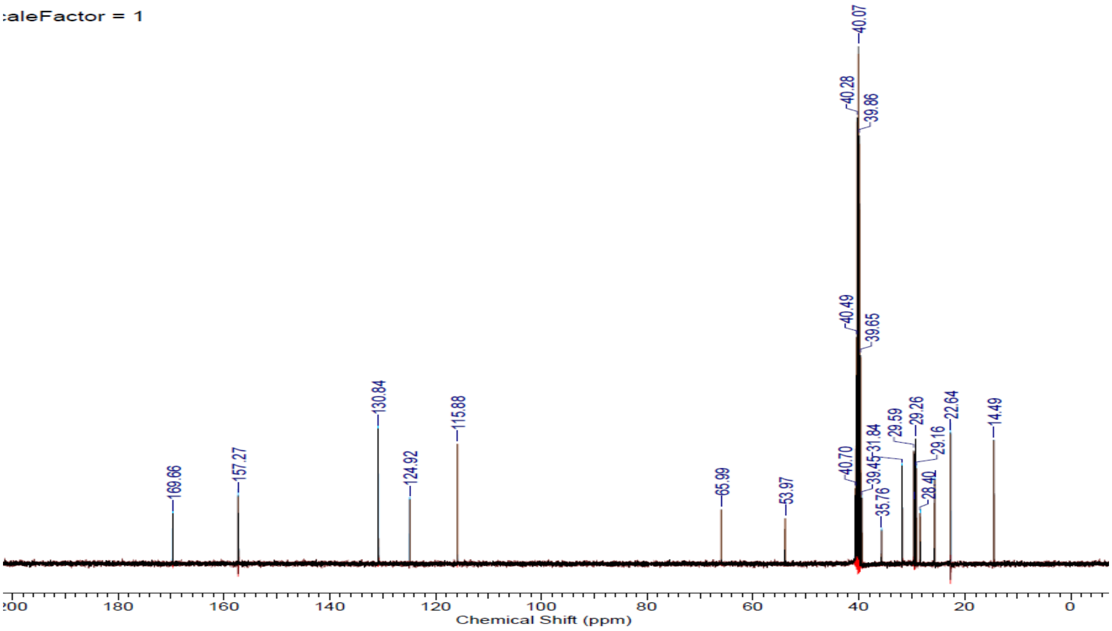

Figure S21. HRMS(FAB+)

Note : m-NBA
Inlet : Direct   Ion Mode : FAB+
RT : 0.00 min   Scan# : (1,25)
Elements : C 100/0, H 100/0, N 3/0, O 10/0
Mass Tolerance   : 1000ppm, 5mmu if m/z < 5, 10mmu if m/z > 10
Unsaturation (U.S.) : -0.5 - 10.0

	Observed m/z	Int%	Err[ppm / mmu]	U.S.	Composition
1	350.2694	15.54	-8.0 / -2.8	9.0	C24 H34 N2
2			+27.9 / +9.8	9.5	C23 H32 N3
3			+24.1 / +8.4	9.0	C25 H34 O
4			-0.3 / -0.1	4.5	C21 H36 N O3
5			-24.8 / -8.7	0.0	C17 H38 N2 O5
6			+11.1 / +3.9	0.5	C16 H36 N3 O5
7			+7.3 / +2.6	0.0	C18 H38 O6

8. N, N-Dimethylglycine ethyl ester hydrochloride (1h)

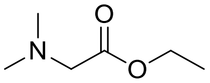

$C_6H_{13}NO_2$

Figure S22. [1]H nmr

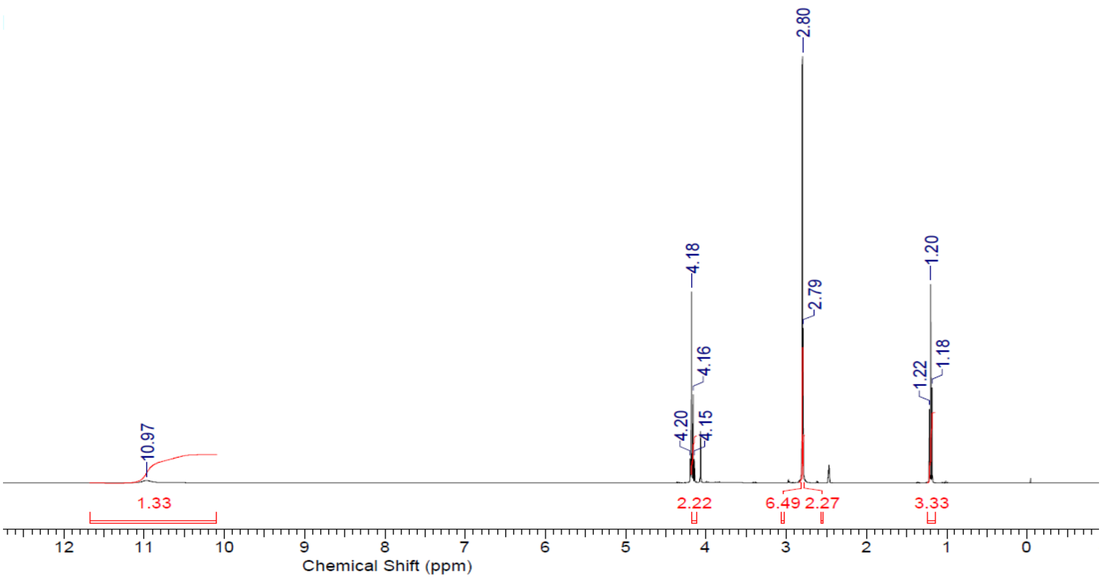

Figure S23. [13]C nmr

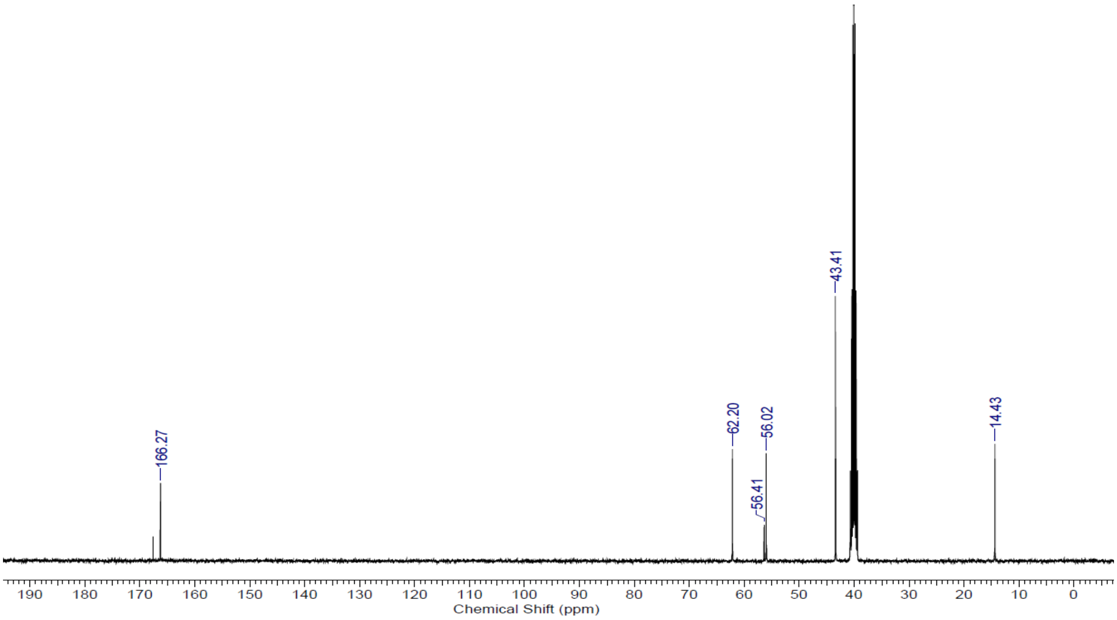

Figure S24. HRMS(FAB+)

Note : m-NBA
Inlet : Direct Ion Mode : FAB+
RT : 0.27 min Scan# : (8,18)
Elements : C 100/0, H 100/0, N 3/0, O 10/0
Mass Tolerance : 1000ppm, 5mmu if m/z < 5, 10mmu if m/z > 10
Unsaturation (U.S.) : -0.5 - 10.0

	Observed m/z	Int%	Err[ppm / mmu]	U.S.	Composition
1	132.1025	58.10	+65.1 / +8.6	5.0	C10 H12
2			+0.3 / +0.0	0.5	C6 H14 N O2

6. Copy of TGA Data

Figure S25. *D*-LAE TGA data

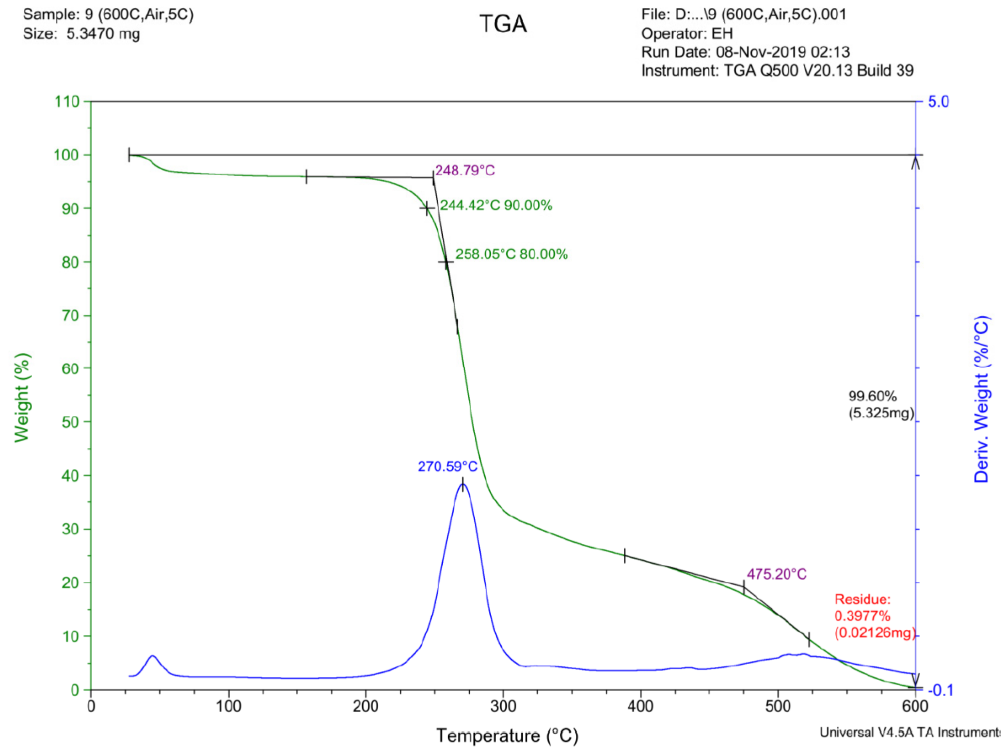

Figure S26. *D*-LAE+AuNP TGA data

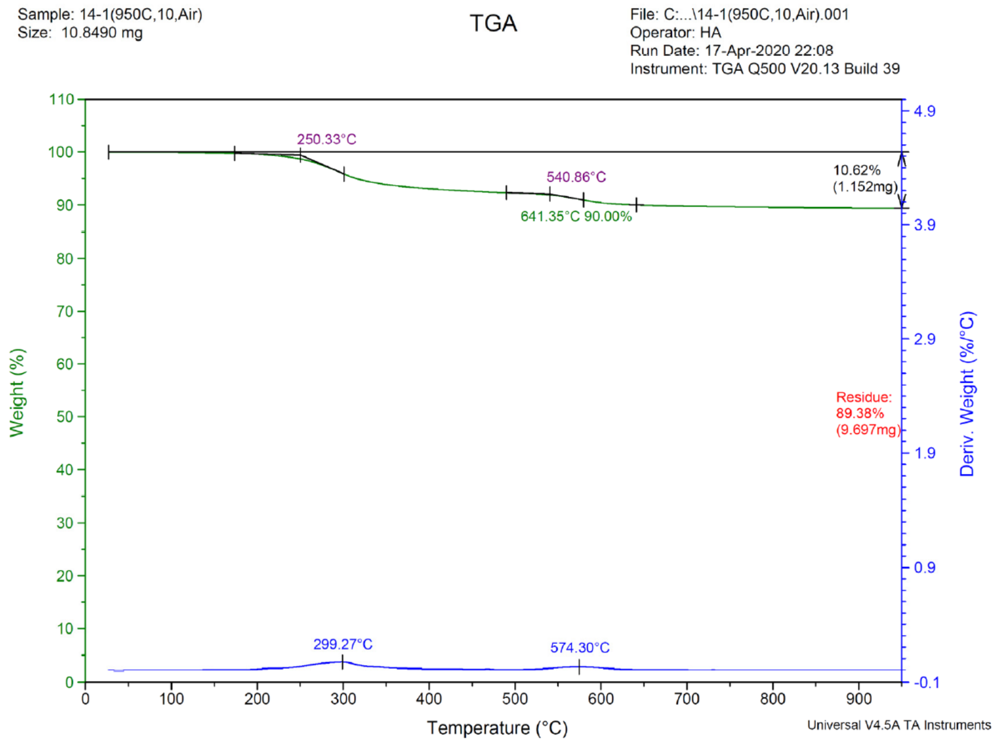

Figure S27. SiNP TGA data

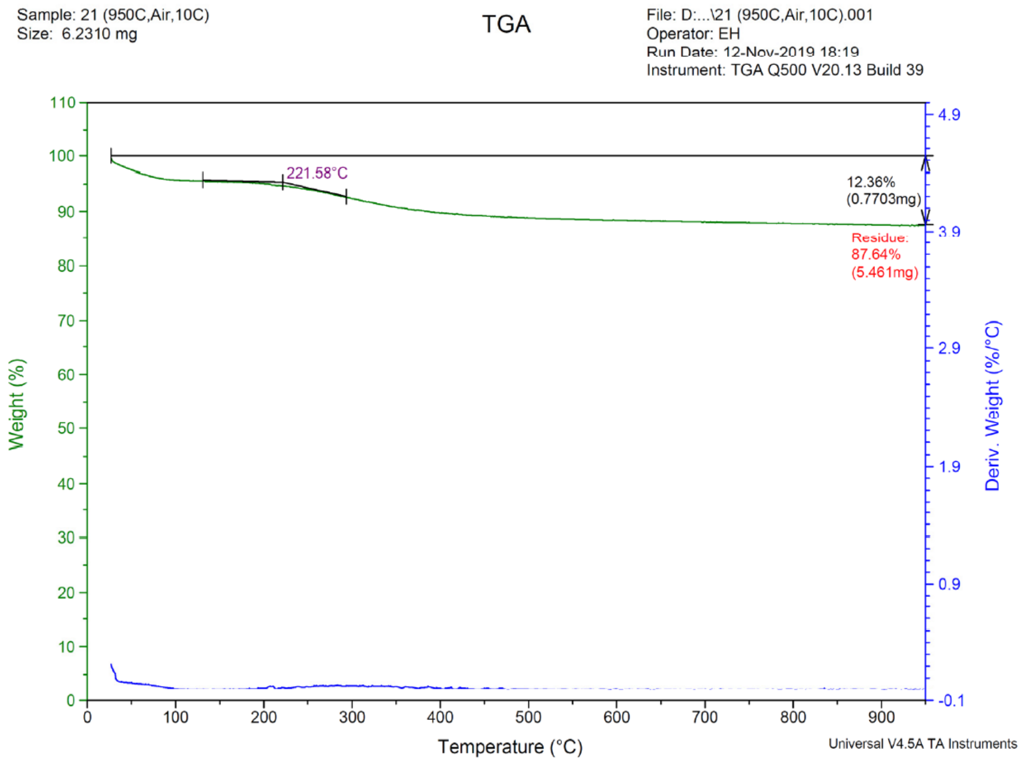

Figure S28. *D*-LAE+SiNP TGA data

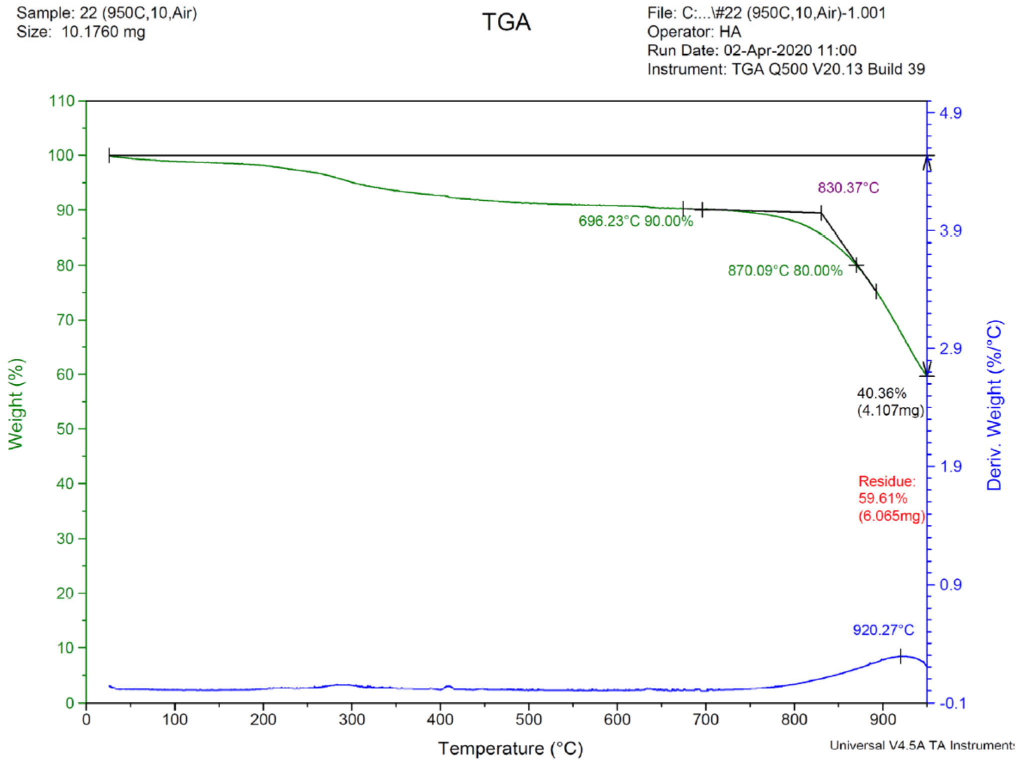

Figure S29. *D*-AD+AuNP TGA data

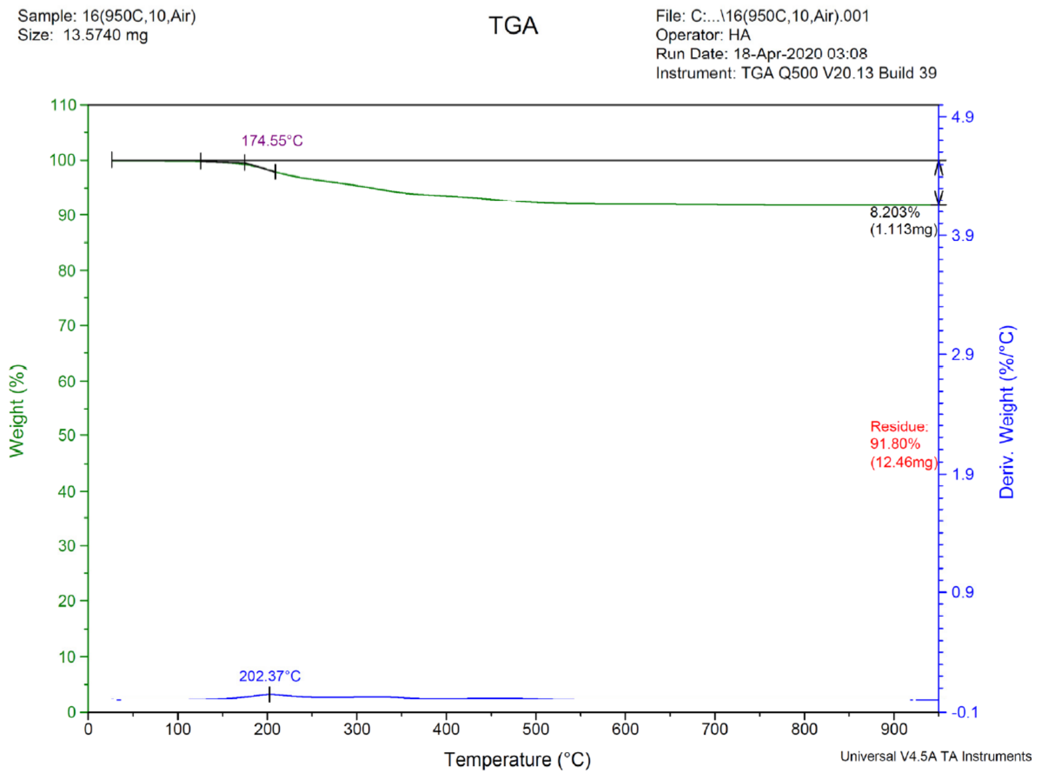

Figure S30. *D*-PDAuNP TGA data

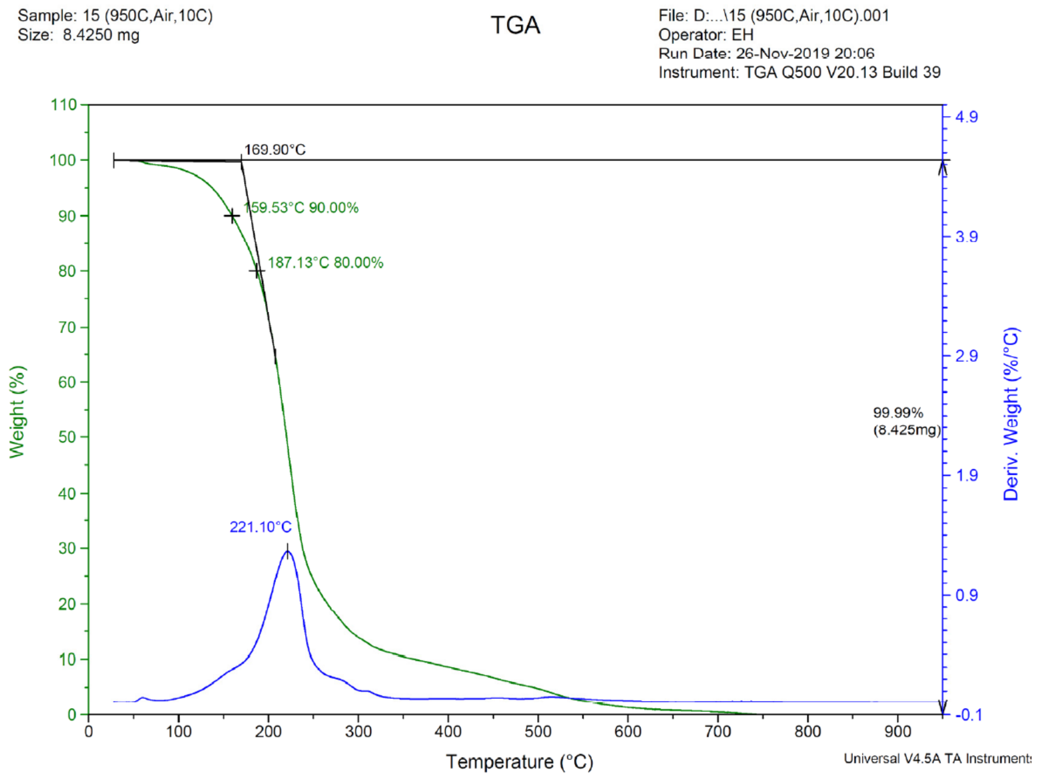

7. Copy of SEM Data

Figure S31. *D*-LAE+AuNP(14)

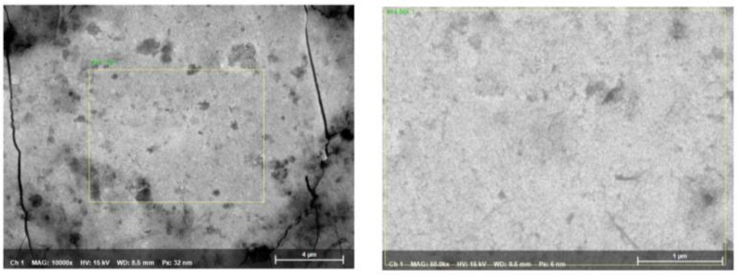

Analysis Element	Unit	Analysis Method	Result			
			Spectrum 1	Spectrum 2	Spectrum 3	Average
C (Carbon)	wt %	SEM-EDX	7.3	24.1	16.6	16.0
N (Nitrogen)	wt %		2.0	3.5	2.5	2.6
O (Oxygen)	wt %		2.0	3.3	2.7	2.6
Al (Aluminium)	wt %		-	0.4	-	0.1
Si (Silicon)	wt %		1.0	-	0.6	0.5
Cl (Chlorine)	wt %		0.1	0.2	0.1	0.1
Au (Gold)	wt %		87.6	68.5	77.6	77.9
**Total**	**wt %**	-	**100.0**	**100.0**	**100.0**	**100.0**

Figure S32. *D*-PD+AuNP(15)

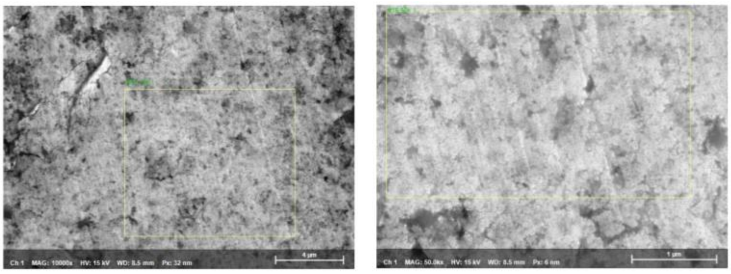

Analysis Element	Unit	Analysis Method	Result			
			Spectrum 1	Spectrum 2	Spectrum 3	Average
C (Carbon)	wt %	SEM-EDX	10.7	19.0	9.4	13.0
N (Nitrogen)	wt %		1.4	1.7	1.2	1.4
O (Oxygen)	wt %		2.8	4.0	2.3	3.0
Si (Silicon)	wt %		1.4	1.2	1.1	1.2
Cl (Chlorine)	wt %		0.5	0.5	0.4	0.5
Au (Gold)	wt %		83.3	73.6	85.7	80.9
**Total**	**wt %**	-	**100.0**	**100.0**	**100.0**	**100.0**

Figure S33. *D*-AD+AuNP(16)

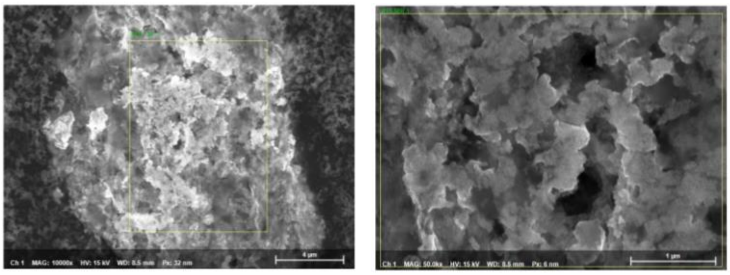

Analysis Element	Unit	Analysis Method	Result			
			Spectrum 1	Spectrum 2	Spectrum 3	Average
C (Carbon)	wt %	SEM-EDX	14.3	19.9	21.9	18.7
N (Nitrogen)	wt %		1.1	0.3	0.2	0.5
O (Oxygen)	wt %		5.7	6.0	7.3	6.3
Na (Sodium)	wt %		0.6	0.7	1.2	0.8
Al (Aluminium)	wt %		0.5	0.8	1.1	0.8
Si (Silicon)	wt %		1.6	2.2	2.2	2.0
P (Phosphorus)	wt %		1.3	1.3	1.0	1.2
Cl (Chlorine)	wt %		0.1	0.2	0.1	0.1
Au (Gold)	wt %		74.8	68.6	65.1	69.5
**Total**	**wt %**	-	**100.0**	**100.0**	**100.0**	**100.0**

Figure S34. SiNP(21)

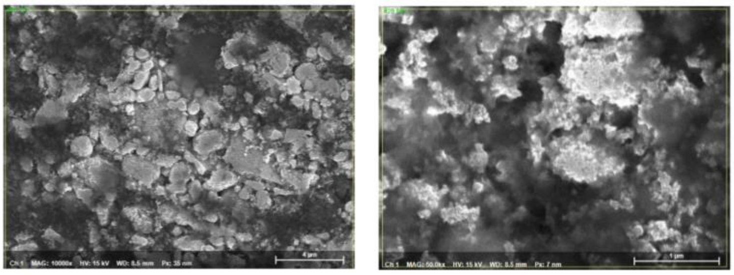

Analysis Element	Unit	Analysis Method	Result			
			Spectrum 1	Spectrum 2	Spectrum 3	Average
C (Carbon)	wt %	SEM-EDX	23.9	36.8	33.4	31.4
N (Nitrogen)	wt %		1.0	1.7	1.6	1.4
O (Oxygen)	wt %		41.5	44.3	46.1	44.0
Na (Sodium)	wt %		1.0	0.5	0.7	0.7
Si (Silicon)	wt %		31.9	16.8	18.2	22.3
Au (Gold)	wt %		0.7	-	-	0.2
**Total**	**wt %**	-	**100.0**	**100.0**	**100.0**	**100.0**

Figure S35. *D*-LAE+SiNP(22)

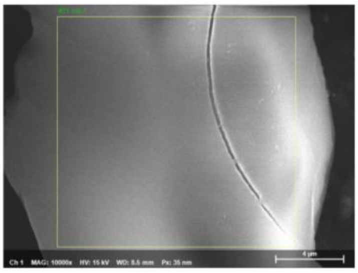

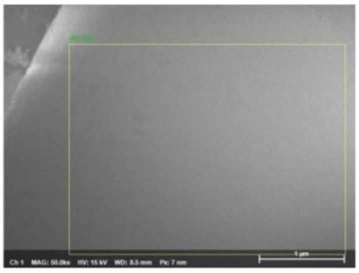

Analysis Element	Unit	Analysis Method	Result			
			Spectrum 1	Spectrum 2	Spectrum 3	Average
C (Carbon)	wt %	SEM-EDX	23.9	36.8	33.4	31.4
N (Nitrogen)	wt %		1.0	1.7	1.6	1.4
O (Oxygen)	wt %		41.5	44.3	46.1	44.0
Na (Sodium)	wt %		1.0	0.5	0.7	0.7
Si (Silicon)	wt %		31.9	16.8	18.2	22.3
Au (Gold)	wt %		0.7	-	-	0.2
**Total**	**wt %**	-	**100.0**	**100.0**	**100.0**	**100.0**

8. Copy of Zeta Potential Analysis Data

Figure S36. *D*-LAE+AuNP

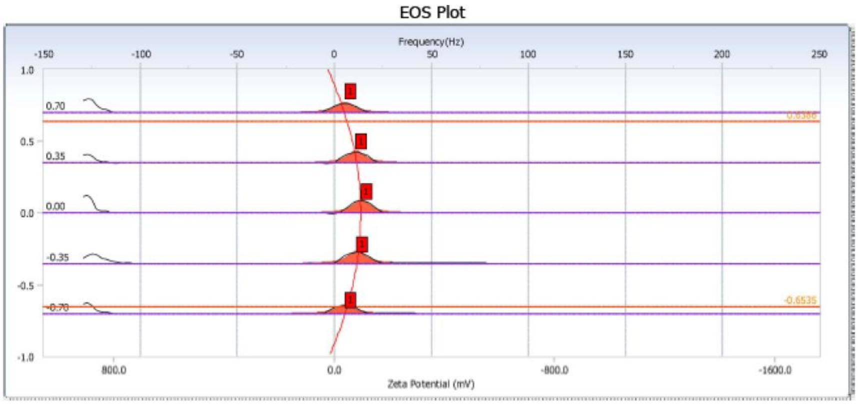

Measurement Results

Zeta Potential	: -47.16	(mV)		Doppler shift	: 6.69	(Hz)
Mobility	: -6.183e-005	(cm²/Vs)		Base Frequency	: 129.5	(Hz)
Conductivity	: 0.0232	(mS/cm)		Conversion Equation	: Huckel	

Zeta Potential of Cell				Diluent Properties		
Upper Surface	: -70.59	(mV)		Diluent Name	: Ethyl Alcohol	
Lower Surface	: -67.06	(mV)		Temperature	: 25.0	(°C)
Cell Condition				Refractive Index	: 1.3611	
Cell Type	: Flow Cell			Viscosity	: 1.1015	(cP)
Avg. Electric Field	: -22.39	(V/cm)		Dielectric Constant	: 24.5	
Avg. Current	: -0.03	(mA)				

Figure S37. *D*-PD+AuNP

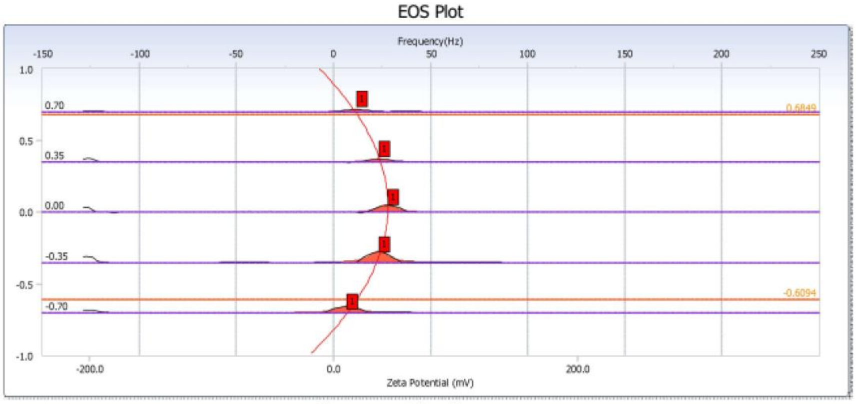

Measurement Results

Zeta Potential	: 19.38	(mV)		Doppler shift	: 12.17	(Hz)
Mobility	: 1.511e-004	(cm²/Vs)		Base Frequency	: 128.7	(Hz)
Conductivity	: 0.0586	(mS/cm)		Conversion Equation	: Smoluchowski	

Zeta Potential of Cell				Diluent Properties		
Upper Surface	: 30.73	(mV)		Diluent Name	: WATER	
Lower Surface	: 39.89	(mV)		Temperature	: 25.0	(°C)
Cell Condition				Refractive Index	: 1.3328	
Cell Type	: Flow Cell			Viscosity	: 0.8878	(cP)
Avg. Electric Field	: 16.67	(V/cm)		Dielectric Constant	: 78.3	
Avg. Current	: 0.04	(mA)				

Figure S38. *D*-AD+AuNP

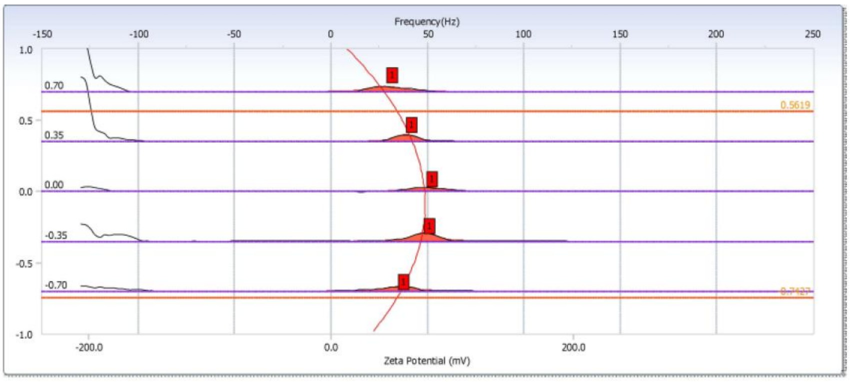

Measurement Results

Zeta Potential	: 54.82	(mV)	Doppler shift	: 34.44	(Hz)
Mobility	: 4.275e-004	(cm²/Vs)	Base Frequency	: 129.6	(Hz)
Conductivity	: 0.0412	(mS/cm)	Conversion Equation	: Smoluchowski	

Zeta Potential of Cell			Diluent Properties		
Upper Surface	: 41.29	(mV)	Diluent Name	: WATER	
Lower Surface	: 21.73	(mV)	Temperature	: 25.0	(°C)
Cell Condition			Refractive Index	: 1.3328	
Cell Type	: Flow Cell		Viscosity	: 0.8878	(cP)
Avg. Electric Field	: 16.68	(V/cm)	Dielectric Constant	: 78.3	
Avg. Current	: 0.03	(mA)			

Figure S39. SiNP Data

EOS Plot

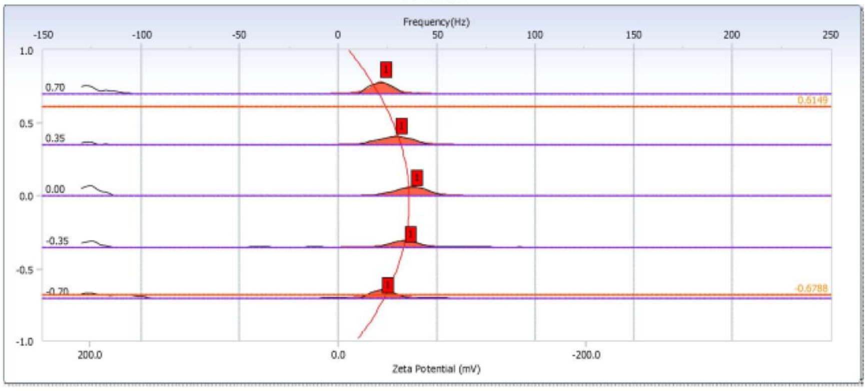

Measurement Results

Zeta Potential	: -38.34	(mV)	Doppler shift	: 24.13	(Hz)
Mobility	: -2.990e-004	(cm²/Vs)	Base Frequency	: 130.2	(Hz)
Conductivity	: 0.0471	(mS/cm)	Conversion Equation	: Smoluchowski	

Zeta Potential of Cell			Diluent Properties		
Upper Surface	: -29.60	(mV)	Diluent Name	: WATER	
Lower Surface	: -23.75	(mV)	Temperature	: 25.0	(°C)
Cell Condition			Refractive Index	: 1.3328	
Cell Type	: Flow Cell		Viscosity	: 0.8878	(cP)
Avg. Electric Field	: -16.71	(V/cm)	Dielectric Constant	: 78.3	
Avg. Current	: -0.03	(mA)			

Figure S40. *D*-LAE+SiNP Data

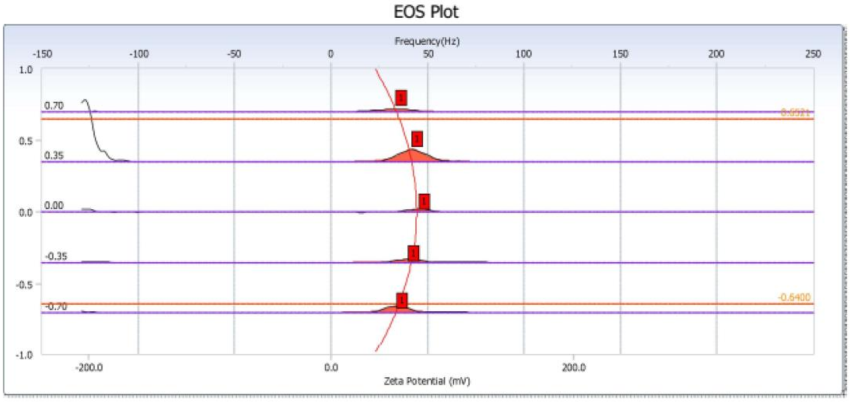

Measurement Results

Zeta Potential	: 56.34	(mV)	Doppler shift	: 35.40	(Hz)
Mobility	: 4.393e-004	(cm^2/Vs)	Base Frequency	: 129.5	(Hz)
Conductivity	: 0.0485	(mS/cm)	Conversion Equation	: Smoluchowski	

Zeta Potential of Cell			Diluent Properties		
Upper Surface	: 19.79	(mV)	Diluent Name	: WATER	
Lower Surface	: 20.63	(mV)	Temperature	: 25.0	(°C)
Cell Condition			Refractive Index	: 1.3328	
Cell Type	: Flow Cell		Viscosity	: 0.8878	(cP)
Avg. Electric Field	: 16.68	(V/cm)	Dielectric Constant	: 78.3	
Avg. Current	: 0.04	(mA)			

9. Computational Results of DFT Calculations for all Calculated Structures

Figure S41. N-*α*-Lauroyl-*D*-Arginine Ethyl ester hydrochloride (1a)

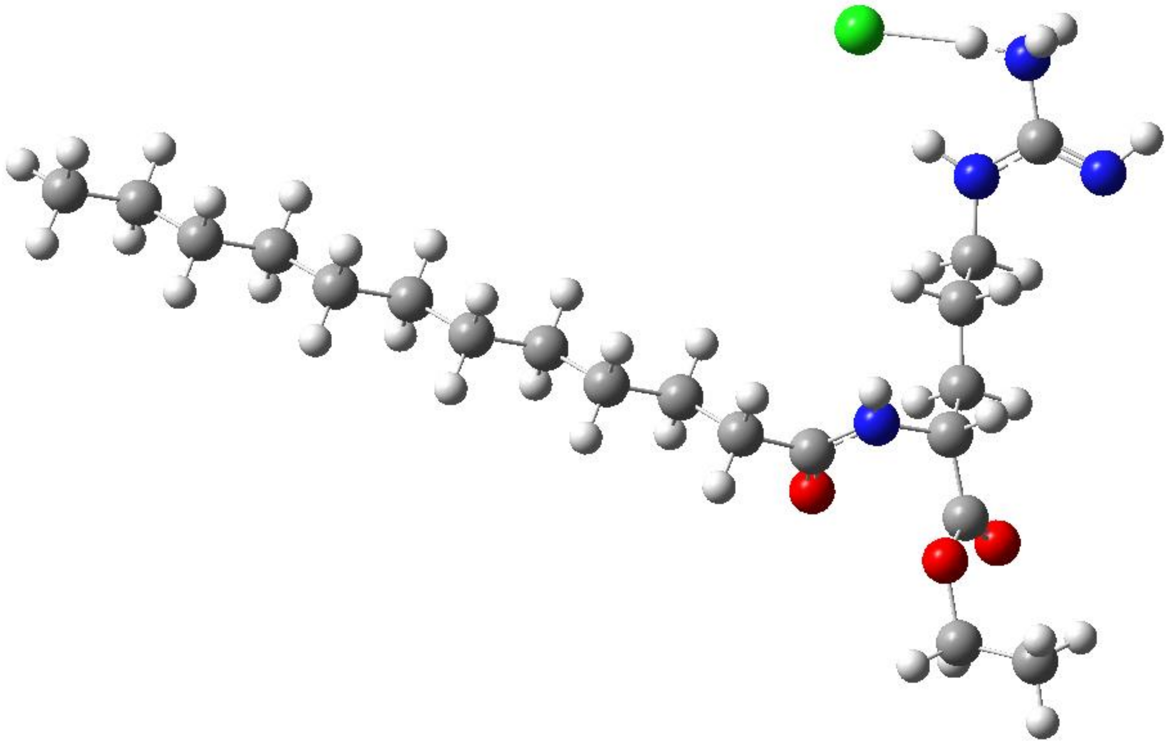

Calculation Type = FREQ

Calculation Method = RB3LYP

Basis Set = 6-311++G(d,p)

Charge = 0

Spin = Singlet

Solvation = scrf=(cpcm,solvent=water)

E(RB3LYP) = -1691.5065 Hartree

Temperature = 298.15 Kelvin

Pressure = 1 atm

Frequencies scaled by = 1

Electronic Energy (EE) = -1691.5065 Hartree

Zero-point Energy Correction = 0.618218 Hartree

Thermal Correction to Energy = 0.653937 Hartree

Thermal Correction to Enthalpy = 0.654881 Hartree

Thermal Correction to Free Energy = 0.539793 Hartree

EE + Zero-point Energy = -1690.8883 Hartree

EE + Thermal Energy Correction = -1690.8525 Hartree

EE + Thermal Enthalpy Correction = -1690.8516 Hartree

EE + Thermal Free Energy Correction = -1690.9667 Hartree

E (Thermal) = 410.352 kcal/mol

Heat Capacity (Cv) = 124.341 cal/mol-kelvin

Entropy (S) = 242.223 cal/mol-kelvin

Symbolic Z-matrix:

Charge = 0 Multiplicity = 1

C	5.17434	-4.09948	-0.19618
N	6.3457	-3.61391	-0.37592
N	4.15391	-3.4976	0.45342
N	4.76298	-5.48241	-0.69542
H	7.01991	-4.20653	-0.86064
H	3.24756	-3.96091	0.49555
C	4.29277	-2.16028	1.04107
H	3.57379	-2.09295	1.86371
H	5.29922	-2.07791	1.46366
C	4.04964	-1.02549	0.02897
H	4.77114	-1.12184	-0.79284
H	3.04715	-1.13932	-0.40197
C	4.18684	0.35451	0.69159
H	3.47655	0.455	1.52006
H	5.19117	0.46814	1.11554

C	3.95353	1.51525	-0.29594
H	4.91925	-5.55459	-1.70612
H	5.32333	-6.20745	-0.23462
N	2.59982	1.5277	-0.86156
C	1.51232	1.93114	-0.14769
O	1.62357	2.32356	1.04721
H	2.48418	1.24153	-1.82235
C	0.17855	1.90577	-0.8724
H	-0.07433	2.94453	-1.13078
H	0.26378	1.35673	-1.81754
C	-0.95211	1.31112	-0.01047
H	-0.71877	0.26213	0.22159
H	-0.98286	1.84598	0.94698
C	-2.32493	1.39118	-0.69989
H	-2.28055	0.86709	-1.66671
H	-2.55566	2.44302	-0.92826
C	-3.46176	0.79346	0.14878
H	-3.22985	-0.25835	0.37624
H	-3.50568	1.31667	1.11629
C	-4.8389	0.87271	-0.53477
H	-5.07184	1.92487	-0.76015
H	-4.79279	0.35233	-1.5039
C	-5.97684	0.27104	0.30973
H	-5.74331	-0.78097	0.53545
H	-6.02355	0.7915	1.27881
C	-7.35377	0.34893	-0.37456
H	-7.58791	1.40106	-0.59913
H	-7.30624	-0.1702	-1.34435
C	-8.49203	-0.25494	0.46804
H	-8.25759	-1.30702	0.69272
H	-8.53987	0.26423	1.43783
C	-9.86871	-0.17774	-0.21654
H	-10.1047	0.8742	-0.44062
H	-9.8217	-0.69645	-1.18671

C	-11.0074	-0.78283	0.62517
H	-10.7708	-1.83368	0.84908
H	-11.0537	-0.2646	1.59453
C	-12.378	-0.7015	-0.06623
H	-12.6534	0.34069	-0.27236
H	-13.1673	-1.13984	0.55584
H	-12.3682	-1.23853	-1.02333
Cl	1.77265	-5.7742	0.03828
H	3.73396	-5.68539	-0.50078
H	4.63478	1.39568	-1.14869
C	4.30884	2.86448	0.33628
O	4.95439	2.99936	1.38516
O	3.91539	3.91292	-0.4442
C	4.22846	5.29388	0.02314
H	3.43708	5.89701	-0.42243
H	4.14187	5.3111	1.11149
C	5.61132	5.71846	-0.44606
H	6.3887	5.10121	0.01376
H	5.78998	6.76181	-0.16246
H	5.69511	5.6405	-1.53442

Figure S42. ***D*-Proline dodecyl ester hydrochloride (1b)**

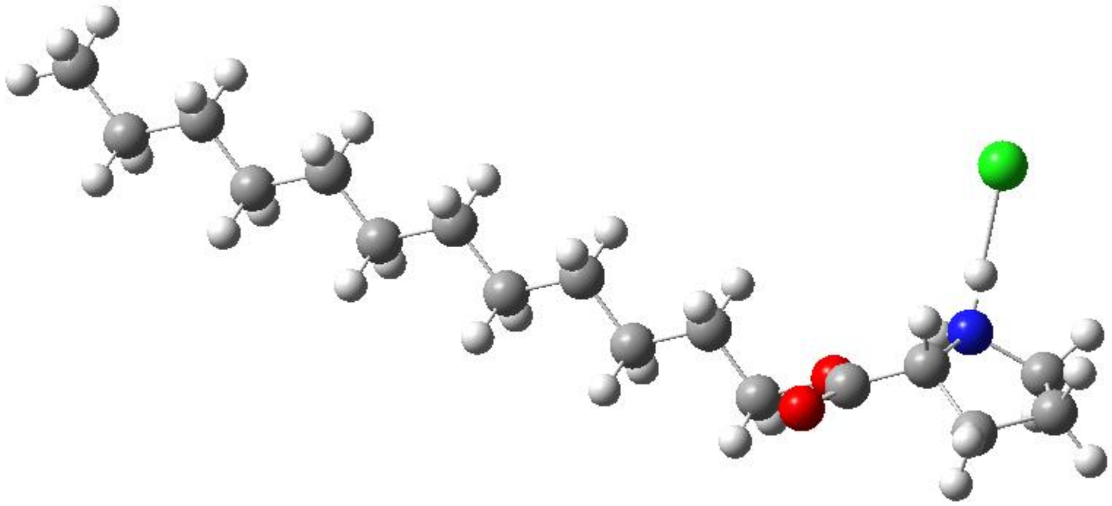

Calculation Type = FREQ

Calculation Method = RB3LYP

Basis Set = 6-311++G(d,p)

Charge = 0

Spin = Singlet

Solvation = scrf=(cpcm,solvent=water)

E(RB3LYP) = -1333.5639 Hartree

Temperature = 298.15 Kelvin

Pressure = 1 atm

Frequencies scaled by = 1

Electronic Energy (EE) = -1333.5639 Hartree

Zero-point Energy Correction = 0.502982 Hartree

Thermal Correction to Energy = 0.5293 Hartree

Thermal Correction to Enthalpy = 0.530244 Hartree

Thermal Correction to Free Energy = 0.438829 Hartree

EE + Zero-point Energy = -1333.0609 Hartree

EE + Thermal Energy Correction = -1333.0346 Hartree

EE + Thermal Enthalpy Correction = -1333.0337 Hartree

EE + Thermal Free Energy Correction = -1333.1251 Hartree

E (Thermal) = 332.141 kcal/mol

Heat Capacity (Cv) = 91.514 cal/mol-kelvin

Entropy (S) = 192.399 cal/mol-kelvin

Symbolic Z-matrix:

Charge = 0 Multiplicity = 1

C	5.66572	-0.30699	0.66202
C	6.88281	-1.27158	0.68611
C	7.96153	-0.51466	-0.1146
C	7.17462	0.14269	-1.25455
H	5.71017	0.38801	1.50516
H	6.62934	-2.21608	0.19234
H	7.18345	-1.49054	1.71219
H	8.4449	0.24562	0.50884
H	8.73614	-1.18372	-0.49741
H	7.62856	1.04826	-1.65698
H	6.9683	-0.55743	-2.0661
H	5.04715	0.33853	-1.23865
N	5.83806	0.51791	-0.60955
C	4.33001	-1.01963	0.70554
O	3.95368	-1.66442	1.69342
O	3.62917	-0.8926	-0.45042
C	2.29342	-1.56064	-0.59764
H	2.30601	-2.46188	0.01895
H	2.26562	-1.82603	-1.65495
C	1.17454	-0.60144	-0.20998
H	1.2685	0.31775	-0.80253
H	1.27724	-0.32363	0.84702

C	-0.21278	-1.23329	-0.44777
H	-0.30392	-2.15435	0.14689
H	-0.3034	-1.52918	-1.50361
C	-1.36851	-0.28065	-0.08846
H	-1.28204	0.01143	0.96932
H	-1.27146	0.6441	-0.67758
C	-2.75858	-0.89563	-0.33448
H	-2.8544	-1.82204	0.25299
H	-2.84254	-1.18771	-1.39289
C	-3.91849	0.0526	0.0221
H	-3.83907	0.33937	1.08239
H	-3.81798	0.98202	-0.56005
C	-5.30906	-0.55723	-0.23512
H	-5.38749	-0.8437	-1.29565
H	-5.40985	-1.48709	0.34639
C	-6.47038	0.38993	0.11985
H	-6.39461	0.67376	1.18128
H	-6.36755	1.32117	-0.45912
C	-7.86067	-0.2182	-0.1435
H	-7.93585	-0.50225	-1.20496
H	-7.96397	-1.14938	0.43552
C	-9.02269	0.72859	0.20983
H	-8.95006	1.01165	1.27183
H	-8.91956	1.66072	-0.36788
C	-10.4132	0.12175	-0.05641
H	-10.4853	-0.16146	-1.11738
H	-10.5162	-0.80969	0.52054
C	-11.5673	1.0748	0.29891
H	-12.5426	0.61458	0.09797
H	-11.5378	1.34879	1.3617
H	-11.5062	2.0015	-0.28672
Cl	5.88951	3.51422	0.21691
H	5.82992	1.54541	-0.36475

Figure S43. ***D*-Alanine dodecyl ester hydrochloride (1c)**

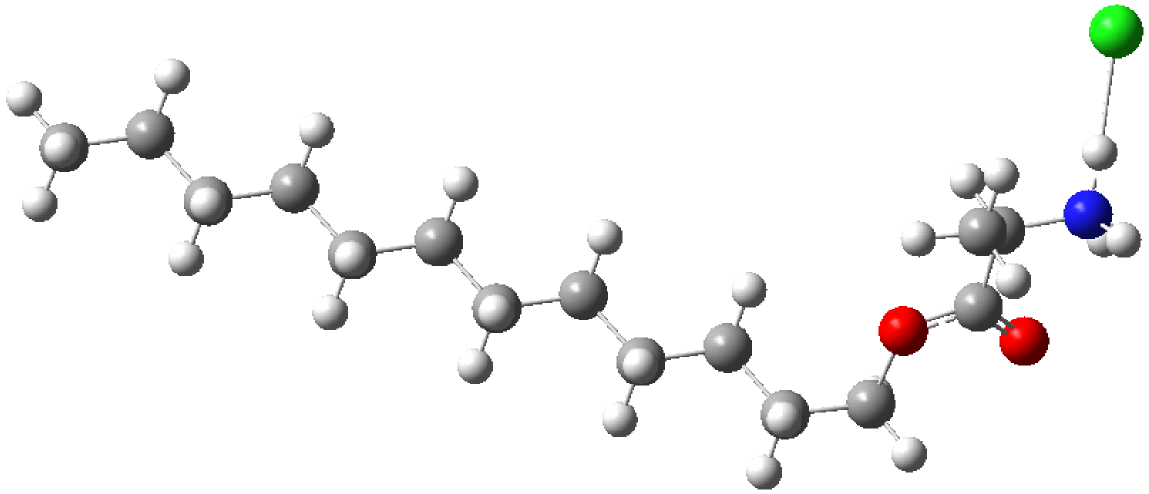

Calculation Type = FREQ

Calculation Method = RB3LYP

Basis Set = 6-311++G(d,p)

Charge = 0

Spin = Singlet

Solvation = scrf=(cpcm,solvent=water)

E(RB3LYP) = -1256.1686 Hartree

Temperature = 298.15 Kelvin

Pressure = 1 atm

Frequencies scaled by = 1

Electronic Energy (EE) = -1256.1686 Hartree

Zero-point Energy Correction = 0.465853 Hartree

Thermal Correction to Energy = 0.491283 Hartree

Thermal Correction to Enthalpy = 0.492227 Hartree

Thermal Correction to Free Energy = 0.40365 Hartree

EE + Zero-point Energy = -1255.7027 Hartree

EE + Thermal Energy Correction = -1255.6773 Hartree

EE + Thermal Enthalpy Correction = -1255.6763 Hartree

EE + Thermal Free Energy Correction = -1255.7649 Hartree

E (Thermal) = 308.284 kcal/mol

Heat Capacity (Cv) = 87.607 cal/mol-kelvin

Entropy (S) = 186.426 cal/mol-kelvin

Symbolic Z-matrix:

Charge = 0 Multiplicity = 1

C	1.62181	-2.75635	0.18501
H	1.64636	-2.71609	1.28234
H	1.28071	-3.76686	-0.08367
C	3.036	-2.61427	-0.34973
H	3.70208	-3.4038	0.00626
H	3.07327	-2.56565	-1.44151
O	3.57843	-1.31159	0.17268
C	4.81296	-0.92844	-0.19026
O	5.57012	-1.57641	-0.93544
C	5.18154	0.43535	0.38241
N	6.63432	0.68836	0.04773
H	6.88635	0.18087	-0.81024
H	6.80879	1.72446	-0.09766
Cl	7.0446	3.72753	-0.41168
H	7.25549	0.35815	0.79261
C	0.62837	-1.71579	-0.36309
H	0.97846	-0.70615	-0.10829
H	0.61095	-1.77666	-1.46211
C	-0.79892	-1.91129	0.18086
H	-1.14998	-2.92343	-0.07377
H	-0.7777	-1.85618	1.2804

C	-1.80264	-0.87538	-0.35834
H	-1.82823	-0.93396	-1.4577
H	-1.44696	0.13647	-0.10869
C	-3.2289	-1.06038	0.19229
H	-3.58555	-2.07182	-0.05798
H	-3.20209	-1.00317	1.29181
C	-4.23245	-0.02245	-0.34371
H	-4.26269	-0.08231	-1.44301
H	-3.87286	0.98893	-0.0972
C	-5.65745	-0.20159	0.21228
H	-6.01772	-1.21281	-0.03387
H	-5.6269	-0.14143	1.31157
C	-6.66044	0.83725	-0.32332
H	-6.69314	0.77539	-1.42247
H	-6.29855	1.8485	-0.07952
C	-8.08461	0.6617	0.23567
H	-8.4478	-0.34935	-0.00741
H	-8.05308	0.72456	1.3349
C	-9.08805	1.7003	-0.30012
H	-9.1198	1.63723	-1.39833
H	-8.72453	2.71044	-0.05819
C	-10.5073	1.51766	0.26471
H	-11.1972	2.27074	-0.13545
H	-10.907	0.52716	0.01053
H	-10.5094	1.60794	1.35894
C	4.93075	0.58103	1.88649
H	5.23723	1.57548	2.22305
H	3.86533	0.46118	2.09116
H	5.48002	-0.17443	2.45765
H	4.605	1.18979	-0.16474

10. Calculated and Experimental FTIR DATA

Figure S44. IR Calculated (N-*α*-Lauroyl-*D*-Arginine Ethyl ester hydrochloride (1a))

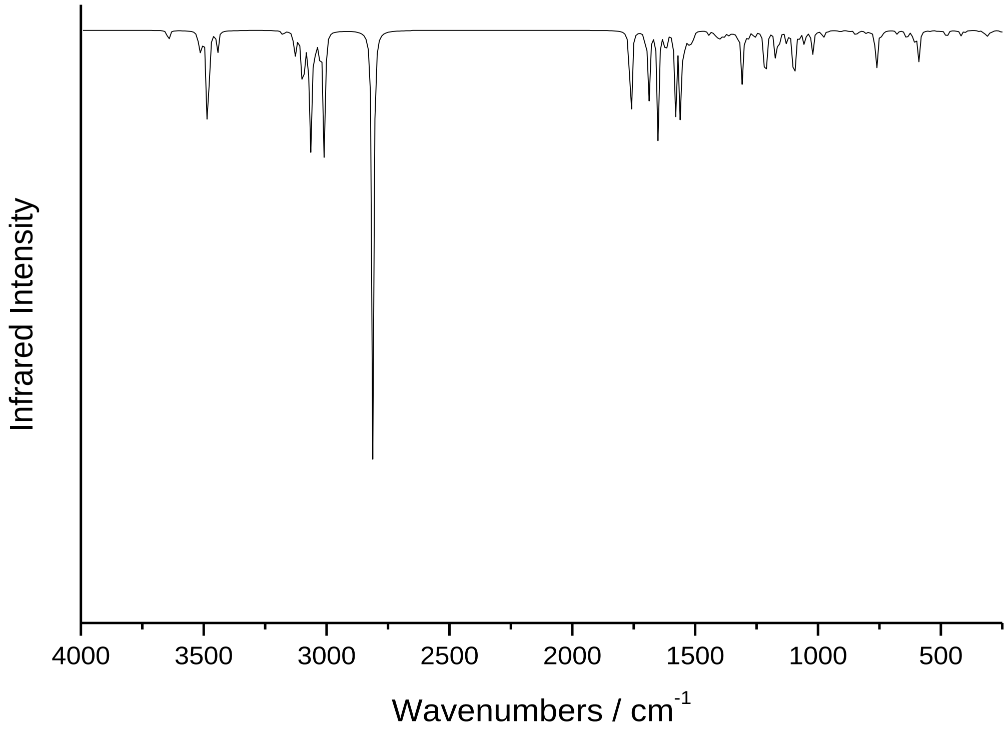

Figure S45. IR Experimental (N-*α*-Lauroyl-*D*-Arginine Ethyl ester hydrochloride (1a))

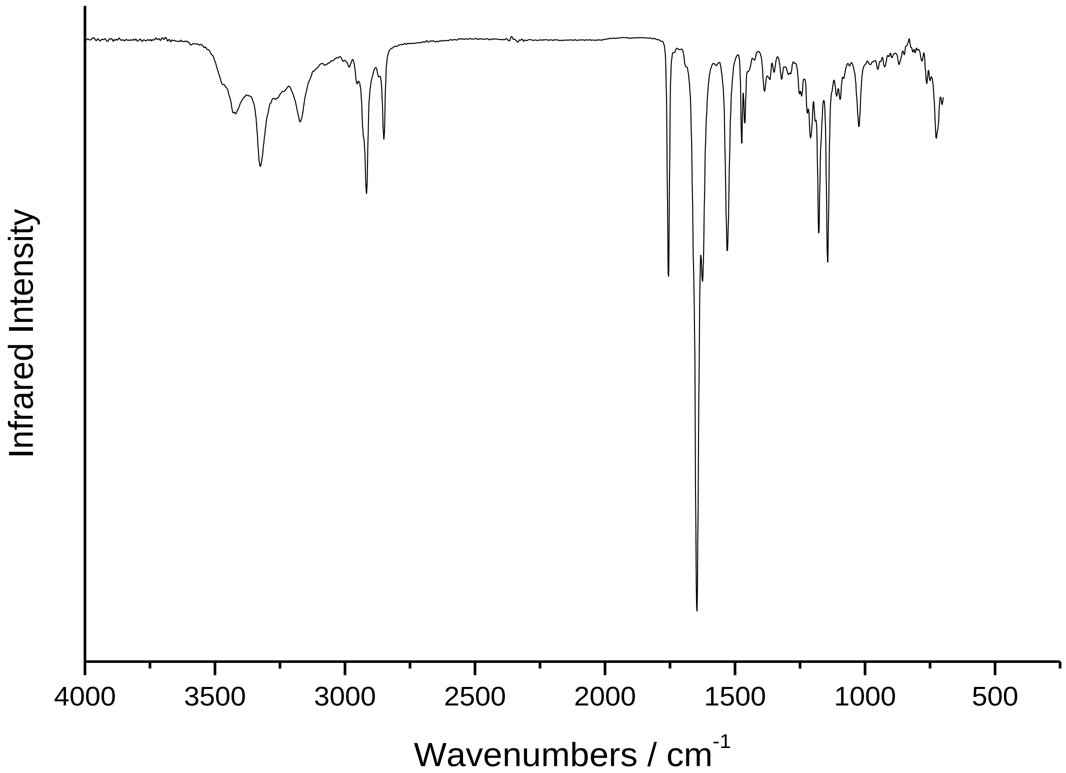

Figure S46. **Raman Calculated (N-*α*-Lauroyl-*D*-Arginine Ethyl ester hydrochloride (1a))**

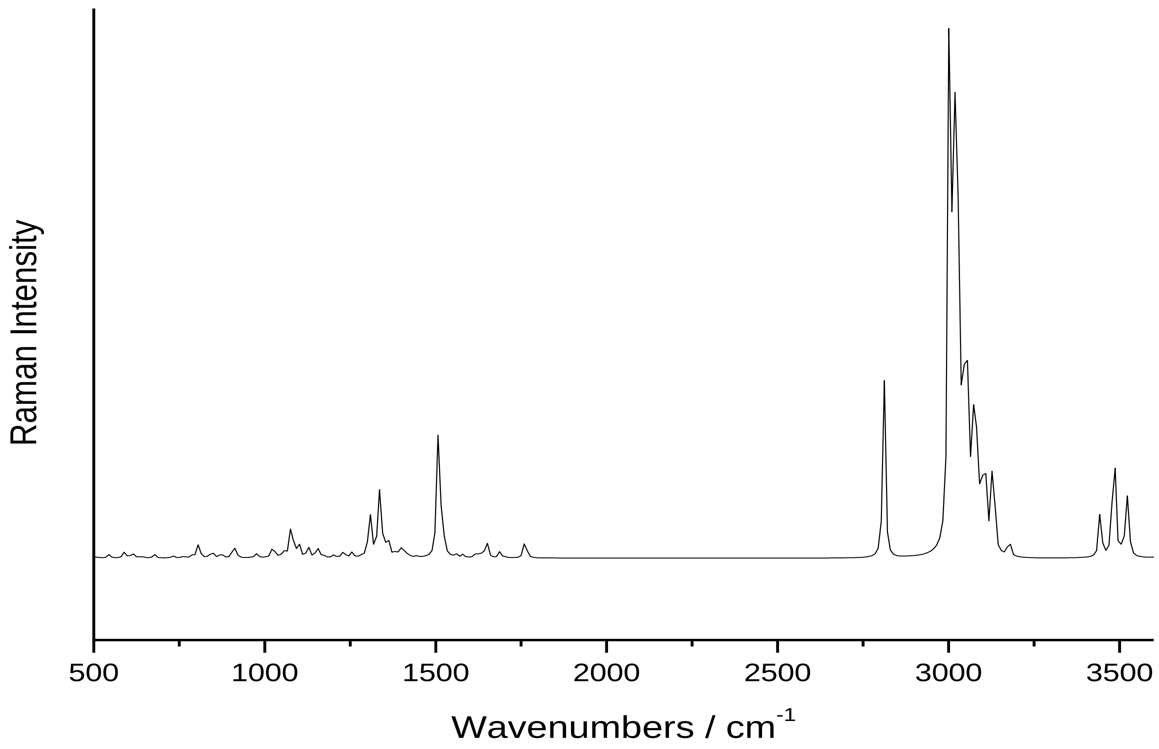

Figure S47. **Raman Experimental (N-*α*-Lauroyl-*D*-Arginine Ethyl ester hydrochloride (1a))**

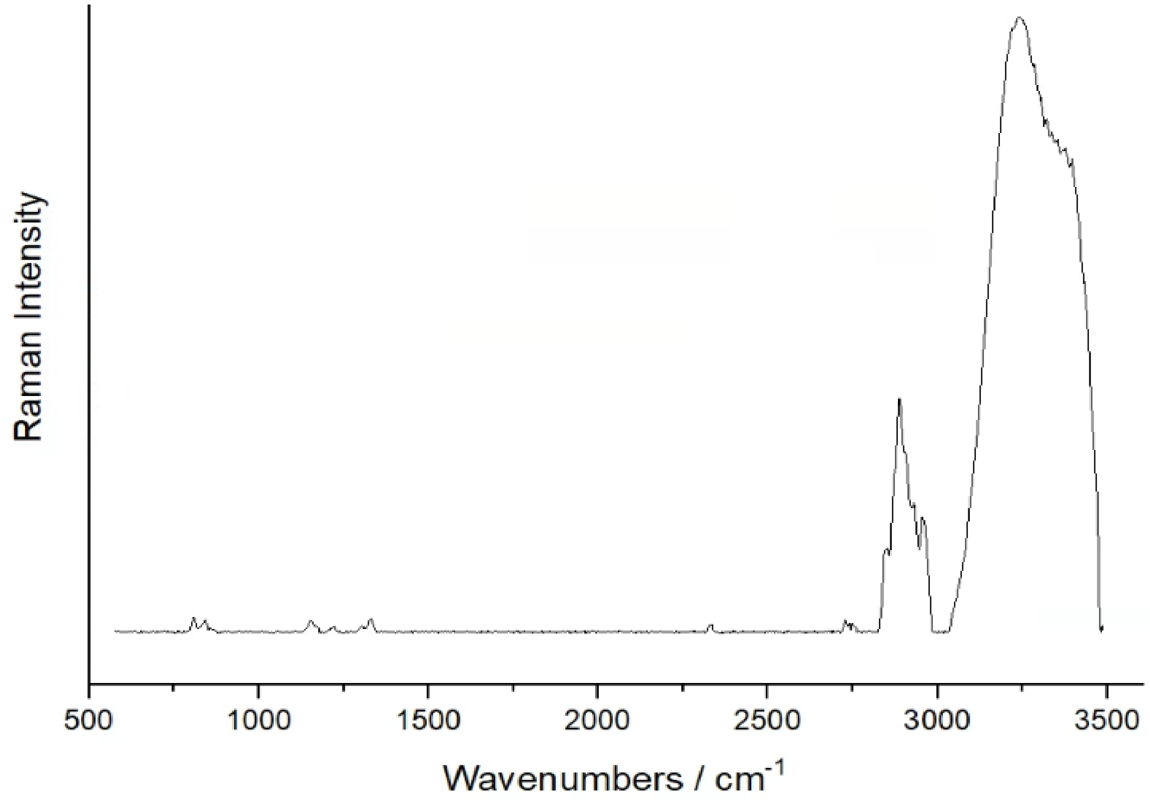

Figure S48. IR Calculated (*D*-Proline dodecyl ester hydrochloride (1b))

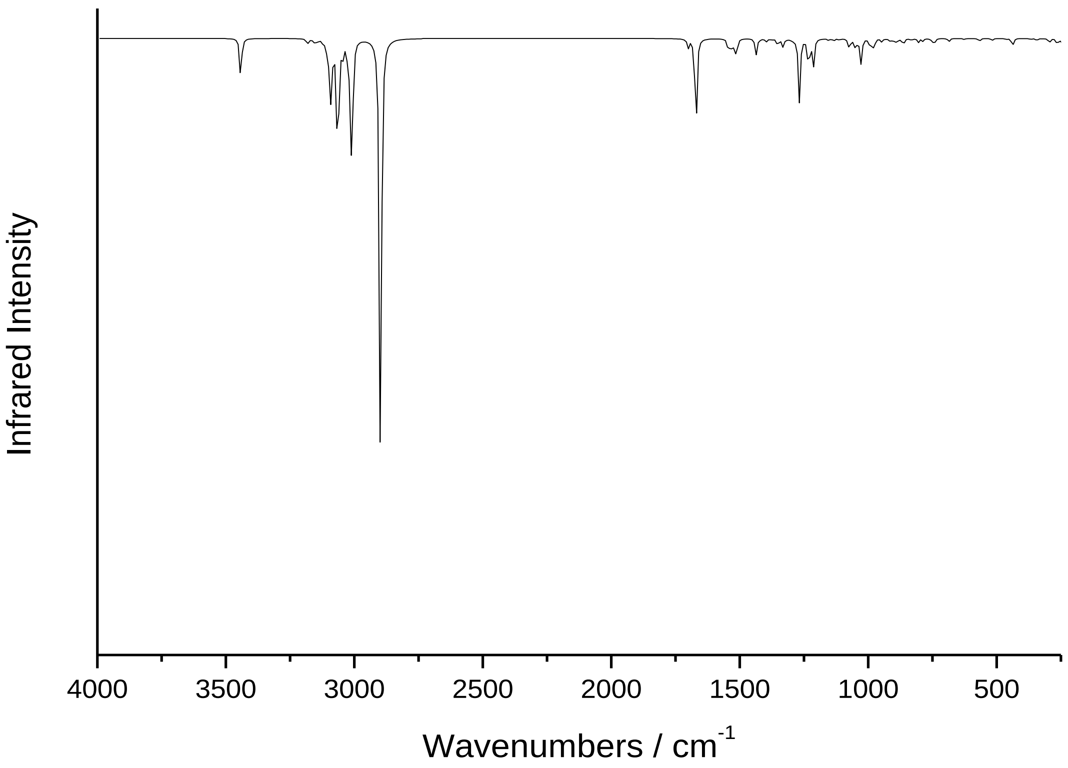

Figure S49. IR Experimental (*D*-Proline dodecyl ester hydrochloride (1b))

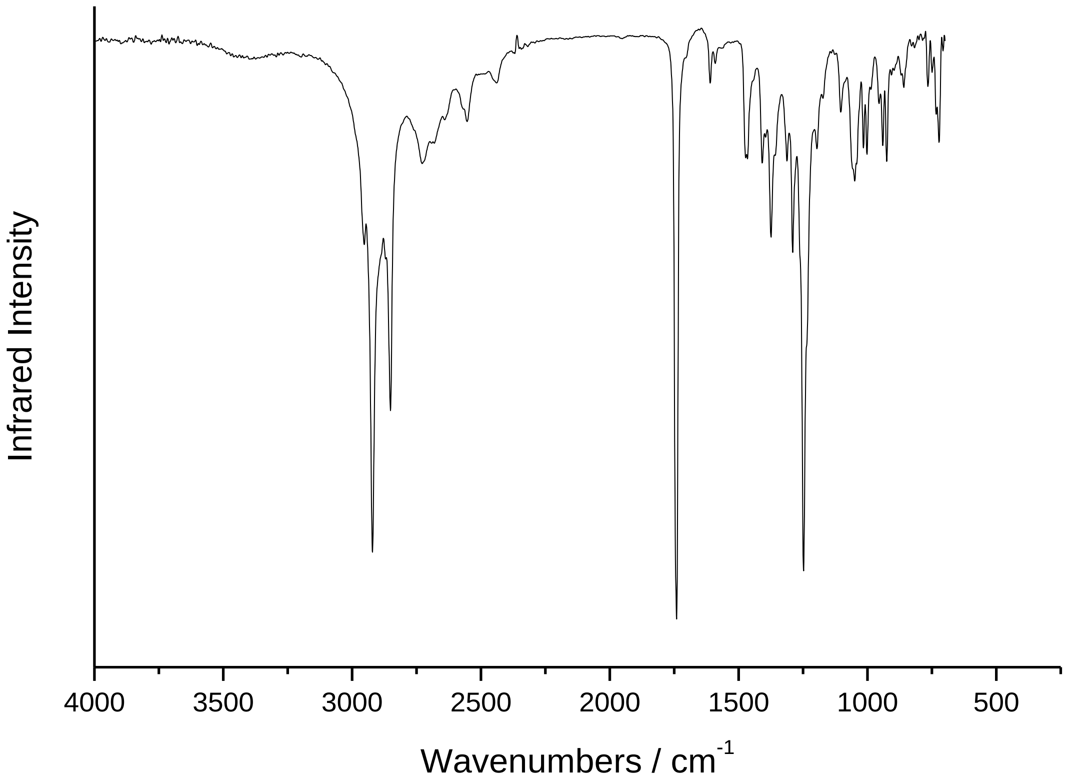

Figure S50. Raman Calculated (*D*-Proline dodecyl ester hydrochloride (1b))

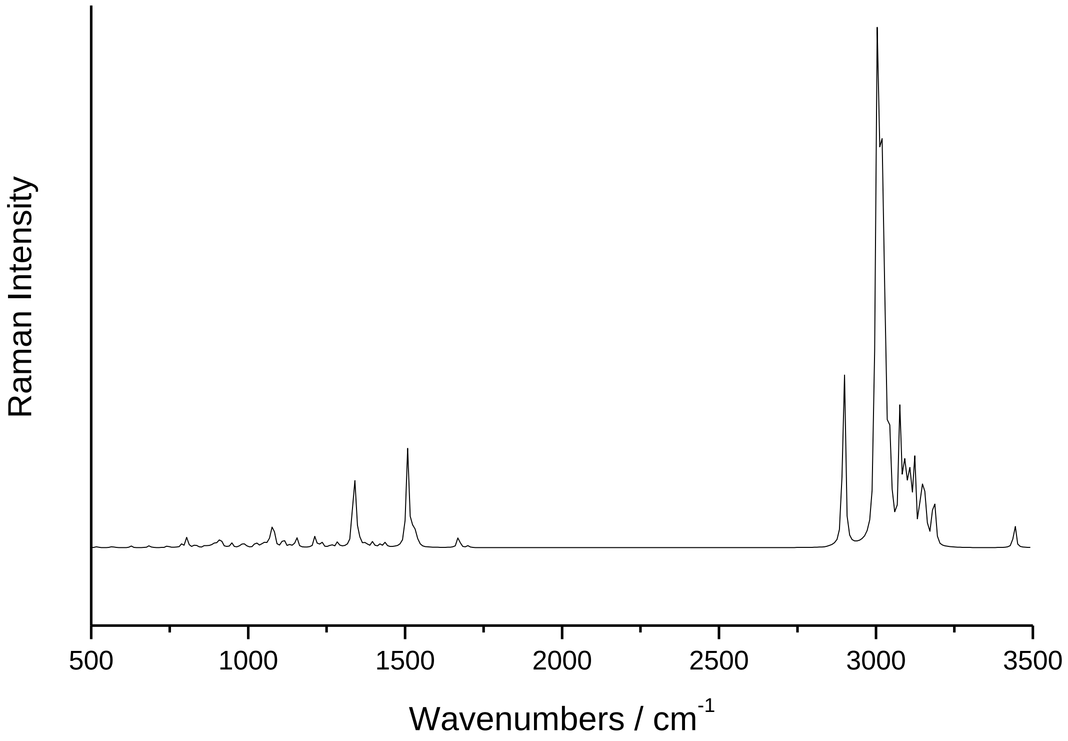

Figure S51. Raman Experimental (*D*-Proline dodecyl ester hydrochloride (1b))

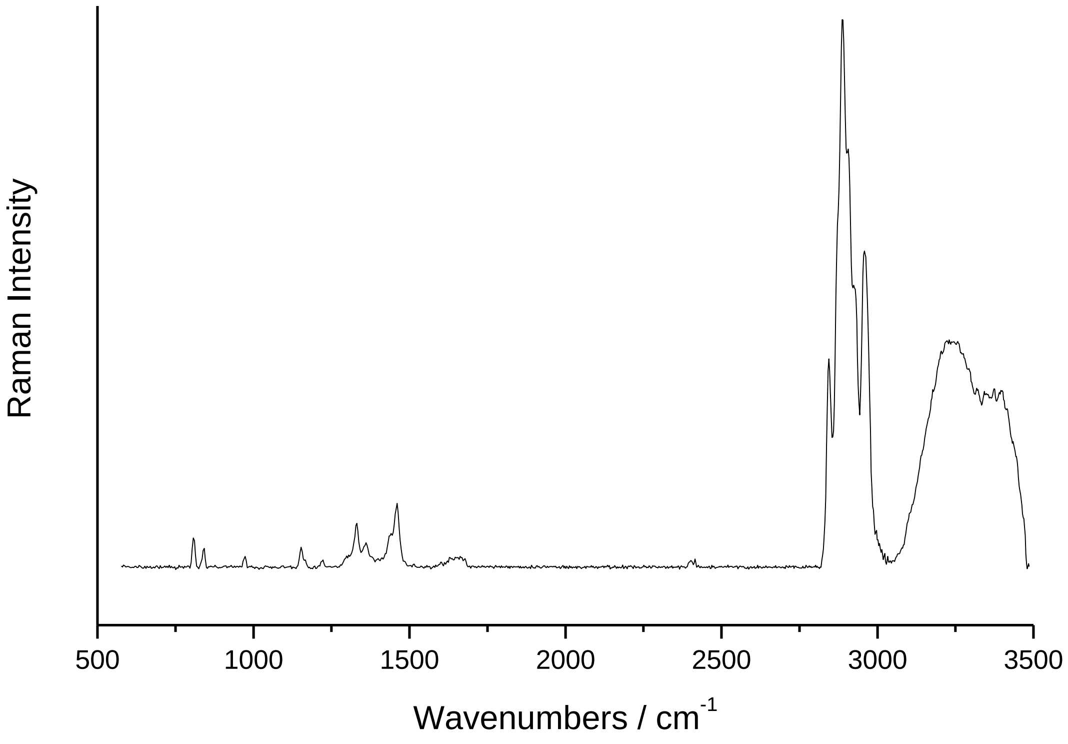

Figure S52. IR Calculated (*D*-Alanine dodecyl ester hydrochloride (1c))

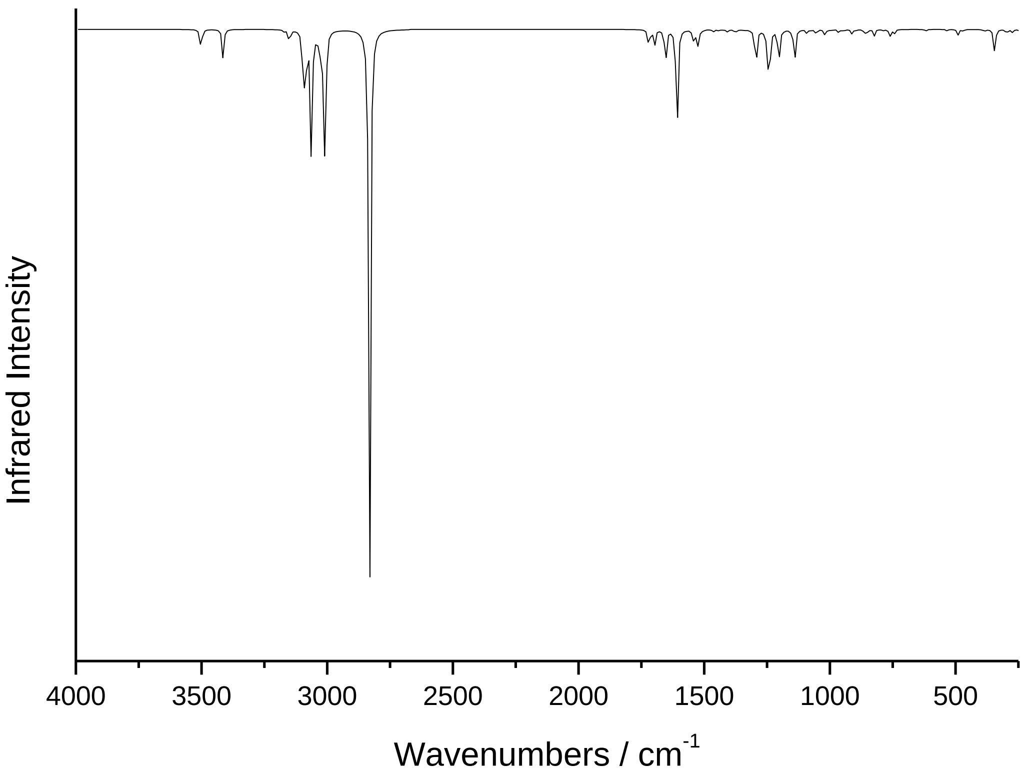

Figure S53. IR Experimental (*D*-Alanine dodecyl ester hydrochloride (1c))

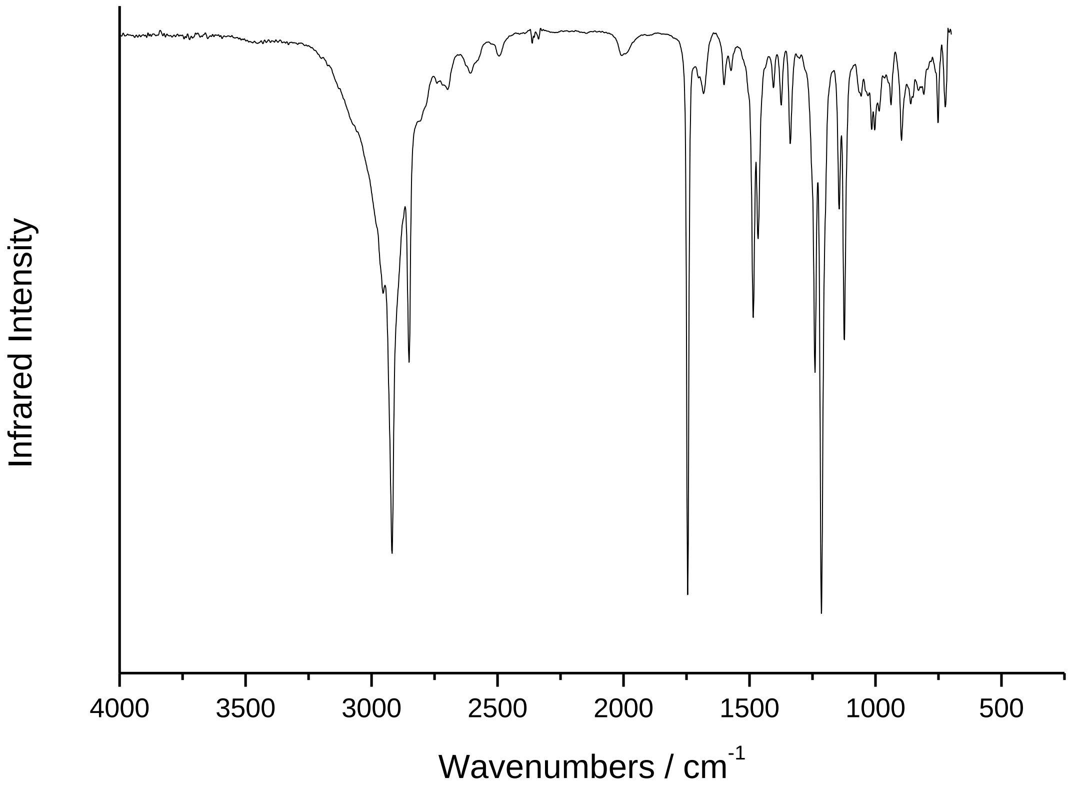

Figure S54. **Raman Calculated (*D*-Alanine dodecyl ester hydrochloride (1c))**

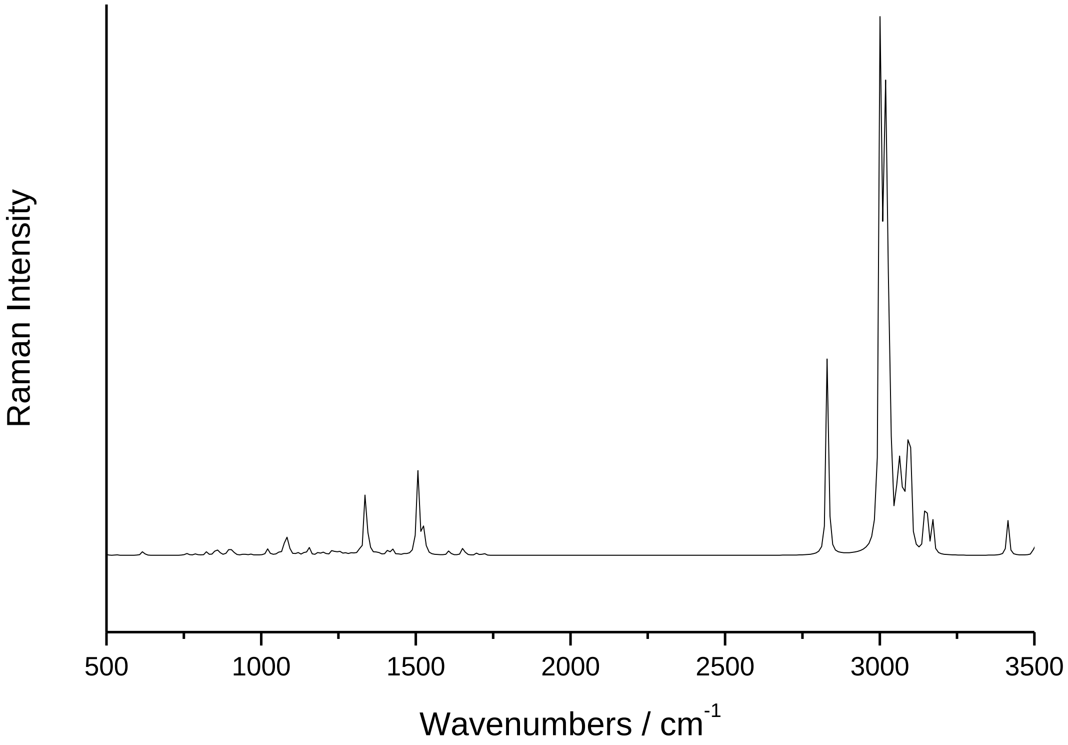

Figure S55. **Raman Experimental (*D*-Alanine dodecyl ester hydrochloride (1c))**

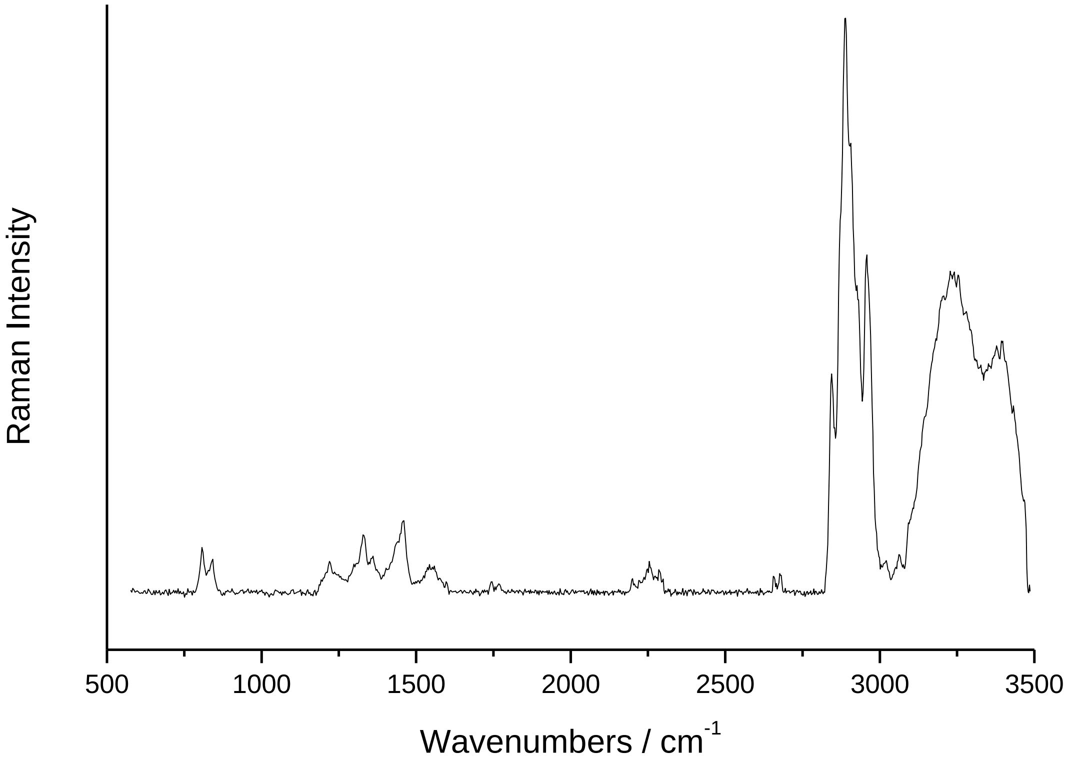

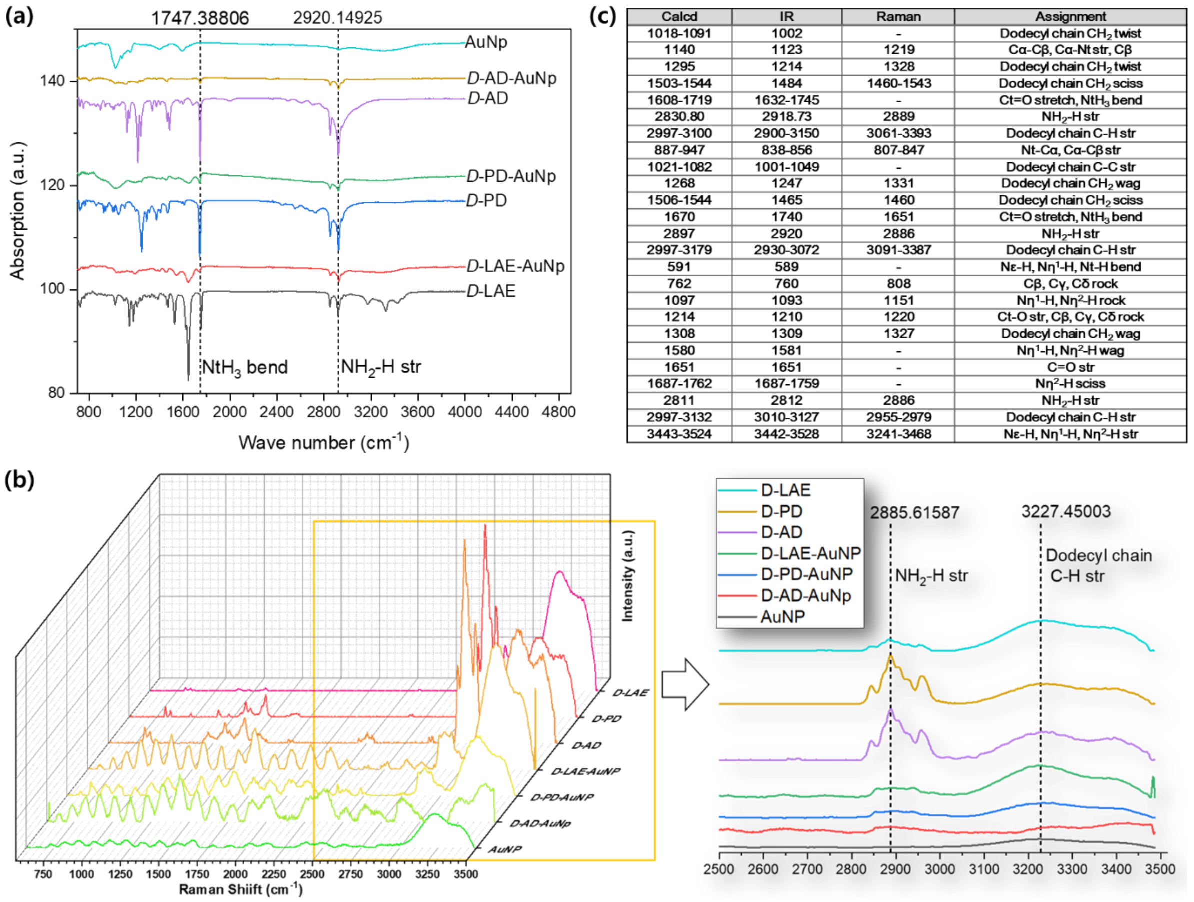

Calcd	IR	Raman	Assignment
1018-1091	1002	-	Dodecyl chain CH_2 twist
1140	1123	1219	Cα-Cβ, Cα-Nt str, Cβ
1295	1214	1328	Dodecyl chain CH_2 twist
1503-1544	1484	1460-1543	Dodecyl chain CH_2 sciss
1608-1719	1632-1745	-	Ct=O stretch, NtH_3 bend
2830.80	2918.73	2889	NH_2-H str
2997-3100	2900-3150	3061-3393	Dodecyl chain C-H str
887-947	838-856	807-847	Nt-Cα, Cα-Cβ str
1021-1082	1001-1049	-	Dodecyl chain C-C str
1268	1247	1331	Dodecyl chain CH_2 wag
1506-1544	1465	1460	Dodecyl chain CH_2 sciss
1670	1740	1651	Ct=O stretch, NtH_3 bend
2897	2920	2886	NH_2-H str
2997-3179	2930-3072	3091-3387	Dodecyl chain C-H str
591	589	-	Nε-H, $N\eta^1$-H, Nt-H bend
762	760	808	Cβ, Cγ, Cδ rock
1097	1093	1151	$N\eta^1$-H, $N\eta^2$-H rock
1214	1210	1220	Ct-O str, Cβ, Cγ, Cδ rock
1308	1309	1327	Dodecyl chain CH_2 wag
1580	1581	-	$N\eta^1$-H, $N\eta^2$-H wag
1651	1651	-	C=O str
1687-1762	1687-1759	-	$N\eta^2$-H sciss
2811	2812	2886	NH_2-H str
2997-3132	3010-3127	2955-2979	Dodecyl chain C-H str
3443-3524	3442-3528	3241-3468	Nε-H, $N\eta^1$-H, $N\eta^2$-H str

Figure S56. (a) IR spectral profiles: Vibrational wavenumbers observed in attenuated total reflection (ATR)-FTIR spectra recorded in H_2O. (b) Raman spectral profiles: Vibrational wavenumbers in Raman spectra recorded in H_2O. (c) Calcd: Calculated results obtained at the DFT/B3LYP/6-311++G(d,p) level on a theoretical model including the AAS in a water solvent. As, intense; m, medium; w, weak; sh, shoulder. Ct and Nt refer to the C and N atoms of the terminal COO^- and NH_3^+ groups, respectively. str, stretching; sciss, scissoring; bend, bending; wag, wagging; rock, rocking; twist, twisting

The Raman and FTIR spectra in H recorded in this study from an experimental viewpoint are presented in Figure S56. The calculated results for the geometry and vibrational modes of the AAS hydrated at the DFT/B3LYP level in a water solvent from a theoretical standpoint are also presented in Figure S56. The estimated number of solvent molecules is sufficient to (i) stabilize the zwitterionic properties of each AAS and (ii) display the appropriate cationic salt form of NH_3^+. Figures S56a and 56b show the differences between the AAS-AuNPs and AAS in the FTIR and Raman spectra. The spectral characteristics of AAS were almost identical to those calculated in Figure 56c. In the FTIR spectra, the peaks at 1747 cm^{-1} and 2920 cm^{-1} were assigned to NtH_3 bending and NH_2–H bonding, respectively. On the other hand, the Raman spectra demonstrated peaks at 2885 cm^{-1} and 3227 cm^{-1} corresponding to NH_2–H bonding and dodecyl chain C–H str, respectively. Both FTIR and Raman spectra indicated the same characteristic bands.

Figure 57. (a) IR spectral profiles(*D*-LAE, SiNP. *D*-LAE+SiNP): Vibrational wavenumbers observed in attenuated total reflection (ATR)-FTIR spectra recorded in water.

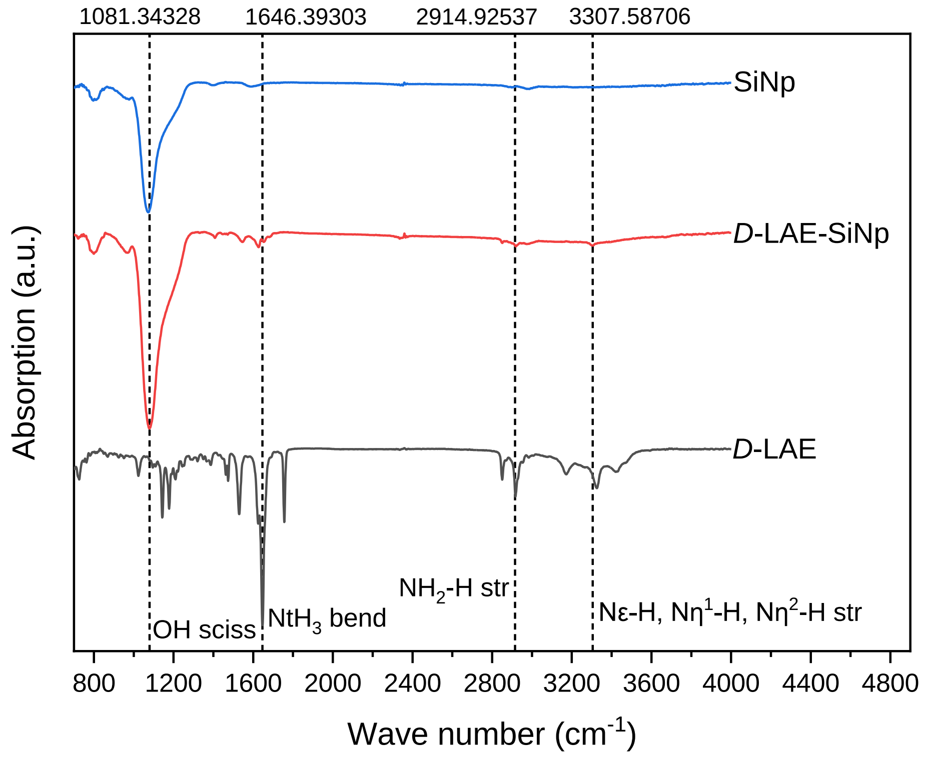

11. Copy of XRD Data

Figure S58. *D*-LAE+SiNP XRD data

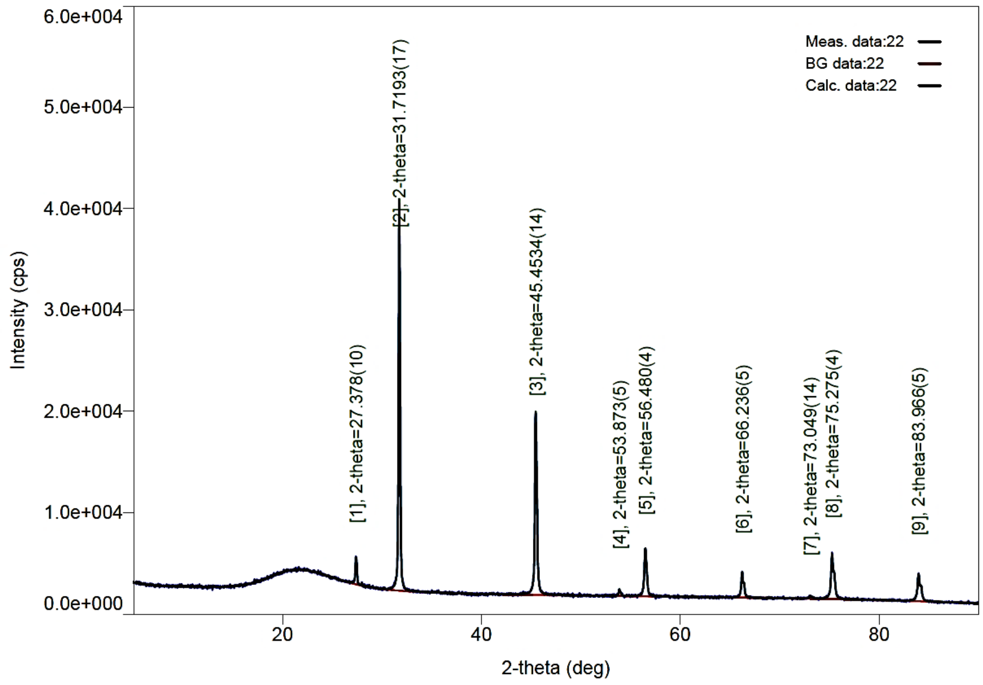

Supplement: Supplementary file 1 — ao2c04220_si_001.pdf [file ao2c04220_si_001.pdf]
